# Different Data for Different Goals: Exploring Trade‐Offs and Synergies in the Use of Spatial Data Inputs to Optimize Conservation Action in Sagebrush Ecosystems

**DOI:** 10.1002/ece3.72214

**Published:** 2025-09-30

**Authors:** Jessica E. Shyvers, Bryan C. Tarbox, Adrian P. Monroe, Nicholas J. Van Lanen, Benjamin S. Robb, Erin K. Buchholtz, Courtney J. Duchardt, David R. Edmunds, Michael S. O'Donnell, Nathan D. Van Schmidt, Julie A. Heinrichs, Cameron L. Aldridge

**Affiliations:** ^1^ U.S. Geological Survey Fort Collins Science Center Fort Collins Colorado USA; ^2^ U.S. Geological Survey South Carolina Cooperative Fish and Wildlife Research Unit, Clemson University Clemson South Carolina USA; ^3^ School of Natural Resources and the Environment University of Arizona Tucson Arizona USA; ^4^ Natural Resource Ecology Laboratory Colorado State University, Fort Collins, Colorado, 80523 in Cooperation with the U.S. Geological Survey, Fort Collins Science Center Fort Collins Colorado USA

**Keywords:** decision‐support tool, ecosystem management, greater sage‐grouse, spatial conservation prioritization, systematic conservation planning, umbrella species

## Abstract

Ecosystems worldwide continue to experience rapid rates of habitat and species loss. Management actions to conserve and restore functional habitats are needed to reduce these declines, but funding and resources for such actions are limited. Spatial conservation prioritization (SCP) can facilitate strategic decision‐making for targeted conservation planning and delivery, but complexities arise when management objectives include multiple wildlife species and ecological or management constraints, all of which can be further complicated by data uncertainty and existing conservation plans. The Prioritizing Restoration of Sagebrush Ecosystems Tool (PReSET), an R package‐based decision‐support tool, supports strategic ecosystem management planning across the sagebrush biome by using SCP. We adapted PReSET to better address the needs of multiple wildlife species, evaluate the effects of different ecological or management constraints on conservation outcomes, assess the influence of data uncertainty, and integrate existing conservation plans. Specifically, we developed optimization problems to identify priority sagebrush protection and restoration across the state of Wyoming, USA, and evaluated the efficacy and trade‐offs of various approaches to problem design. We evaluated trade‐offs in targeting multiple species compared to a single species, including using greater sage‐grouse as a potential umbrella species to benefit other sagebrush‐dependent wildlife. We then evaluated multi‐species protection and restoration problems aimed at minimizing the risks of inadequate connectivity, climate change, and restoration failure, and accounted for data uncertainty to assess relationships between risk aversion of managers and conservation outcomes. We also developed optimization problems within conservation areas identified by an existing sagebrush conservation plan to evaluate the efficacy of guiding local‐scale conservation delivery within more broadly defined conservation areas. Our results demonstrate how SCP methods can leverage novel spatial data to develop targeted decision‐support resources that can facilitate landscape conservation planning and improve management outcomes across a wide array of systems and species.

## Graphical abstract

Management actions to conserve and restore functional habitats are needed to reduce habitat loss and species declines, but funding and resources for such actions are limited. The Prioritizing Restoration of Sagebrush Ecosystems Tool (PReSET) supports strategic ecosystem management planning across the sagebrush biome by using spatial conservation prioritization. We adapted PReSET to better address the needs of multiple wildlife species, evaluate the effects of different ecological or management constraints on conservation outcomes, assess the influence of data uncertainty, and integrate existing conservation plans.

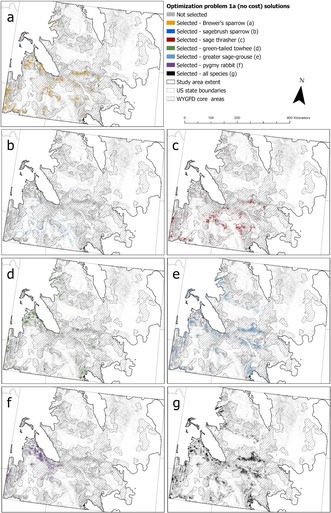

## 1 Introduction

Protecting biodiversity during what is widely considered a sixth mass extinction (Barnosky et al. 2011; Dirzo et al. 2014; Ceballos et al. 2015) is a formidable global challenge, especially as extinction rates are projected to intensify due to ongoing threats including climate change, invasive species, and land‐use change (Urban 2015; Pyšek et al. 2020; Sonter et al. 2020). Anthropogenic disturbances have collectively reduced, fragmented, and degraded ecosystems, resulting in the accelerating and dramatic loss of populations and species (Dirzo et al. 2014; Pimm et al. 2014; Ceballos et al. 2020) and the decline of ecosystem function and services (Hooper et al. 2005; Cardinale et al. 2012; Rosenberg et al. 2019). Addressing these declines and averting the collapse of vital ecosystem processes is possible (Langhammer et al. 2024), but will require urgent and strategic action to protect and restore ecosystems at landscape scales (Leclère et al. 2020; Strassburg et al. 2020). However, limited resources exist to implement these actions, leading to a critical need to allocate resources effectively and efficiently as we endeavor to balance growing human resource demands with maintenance of ecosystem function and species persistence (Moilanen et al. 2009; Leclère et al. 2020).

The discipline of systematic conservation planning was developed to provide a quantitative framework for making conservation decisions in an informed, transparent, and repeatable way (Margules and Pressey 2000), an important component of which is spatial conservation prioritization (hereafter, SCP). SCP is focused specifically on the spatial optimization of conservation actions to promote efficient allocation of resources for mitigating biodiversity loss (Kukkala and Moilanen 2013) by synthesizing data on species diversity, ecological resources, land use, and other socio‐environmental variables (Sarkar et al. 2006). Most commonly, SCP software is used to select a combination of land parcels that meet conservation objectives (e.g., preserving focal species' habitat) while minimizing cost, either as dollar amounts or other variables representing constraints to conservation action (e.g., restoration feasibility, connectivity; Possingham et al. 2000). The flexible nature of SCP allows developers to integrate disparate data to generate customized optimization solutions for a wide array of conservation challenges. This approach has been used to design and establish new protected areas (Lessmann et al. 2014; Li et al. 2020), identify critical ecological corridors (Jalkanen et al. 2019), and optimize trade‐offs between ecosystem services and resource extraction (de Assis Barros et al. 2022).

Despite the demonstrated utility of SCP, major challenges remain regarding its implementation and efficacy (Wiersma and Sleep 2016; McIntosh et al. 2017). Most SCP efforts focus on a single species or biodiversity feature (Wiersma and Sleep 2016), limiting their capacity to accommodate the needs of other coexisting species of conservation concern. Designing SCP problems that integrate the needs of multiple species can involve trade‐offs regarding unique habitat conditions, incongruent distributions, and contrasting spatial scales at which these needs must be met (Duchardt et al. 2021; de Zwaan et al. 2022). Similarly, SCP developers may be interested in optimizing conservation solutions to meet a variety of potential ecological constraints (e.g., connectivity, climate change), which can be complicated even when focusing on a single species (e.g., Dilts et al. 2016). The complexity of synthesizing multiple potential focal species, costs, or other constraints is further exacerbated by uncertainty, which is rarely addressed explicitly in SCP (Wiersma and Sleep 2016). These methodologies involve integrating various datasets with multiple types of error and uncertainty, but without assessments of such uncertainty, end‐users cannot interpret how results of a prioritization solution align with their degree of risk aversion (Eaton et al. 2019). Finally, integrating new research into existing management paradigms is a persistent challenge in adaptive management (Gosselin 2009; Månsson et al. 2023). When conservation practitioners are already working with existing plans and prioritization strategies, it may be unclear how to integrate the results of a new SCP.

The sagebrush (*Artemisia* spp.) biome in the state of Wyoming, USA, provides unique opportunities to explore potential solutions to these conservation planning challenges. Across western North America, over half of the sagebrush biome's historic extent has been lost or degraded (Miller et al. 2012; Rigge, Meyer, and Bunde 2021), and these trends continue, with a 26% decrease in sagebrush cover estimated from 1985 to 2018 (Rigge et al. 2020). The ecological integrity of Wyoming's sagebrush ecosystems, while high, has nevertheless declined since 2001 (Doherty et al. 2022). These ecosystems face continued threats from human development, wildfire, climate change, and invasive species (Davies et al. 2011; Wyoming Game and Fish Department 2017), presenting a needed opportunity to preserve a functioning core of the sagebrush biome. Long‐term persistence of these systems and the species that depend on them will require both protection of intact sagebrush sites and restoration of degraded sites, including areas where connectivity has been lost. Of particular interest is the high‐profile greater sage‐grouse ( *Centrocercus urophasianus* ), which has experienced substantial restrictions in historical range, as well as population declines (Coates et al. 2023). Greater sage‐grouse is a top conservation priority across the western United States (Coates et al. 2023), with Wyoming comprising a critical core of its range (Coates et al. 2021). The species has frequently been proposed as an umbrella species for a suite of sagebrush‐associated species of concern, but assessments on the effectiveness of sage‐grouse conservation in providing overlapping benefits for other species have produced mixed results (Carlisle et al. 2018; Barlow et al. 2019; Runge et al. 2019; Smith et al. 2021; Duchardt et al. 2023; Aldridge et al. 2024). More investigation is needed to determine whether conservation efforts to recover sage‐grouse will provide sufficient protections for other sagebrush‐obligate and associated species of conservation concern compared to a multi‐species approach. Managers of the sagebrush biome therefore face a multitude of considerations when planning habitat protection and restoration actions (Wyoming Game and Fish Department 2020), such as competing wildlife management goals (Van Lanen, Shyvers, et al. 2023), the need to improve habitat connectivity (Tack et al. 2019), long‐term persistence of sagebrush under future climate conditions (Rigge, Shi, and Postma 2021), and feasibility of restoring disturbed sagebrush sites (Chambers et al. 2019), with each factor influencing decisions on where to best allocate limited management resources. Furthermore, state, federal, and non‐governmental agencies and organizations developed independent and joint conservation plans (e.g., Wyoming Game and Fish Department 2020; Doherty et al. 2022), raising questions about how best to integrate updated decision‐support tools.

To support strategic ecosystem management planning efforts across the imperiled sagebrush steppe in Wyoming, USA, we developed an improved iteration of a preliminary R package (R Core Team 2021) based SCP tool, the Prioritizing Restoration of Sagebrush Ecosystems Tool (PReSET; Duchardt et al. 2021). Our expanded tool leverages emerging spatial data resources to provide a structured but customizable set of optimization problems that can guide broad‐scale planning efforts by prioritizing protection and restoration of sagebrush ecosystems. We use the results of these optimization problems to explore the feasibility of balancing conservation of multiple species while minimizing conservation investments and evaluate how including different ecological constraints (e.g., connectivity, long‐term climate change risk, restoration feasibility) and incorporating uncertainty into SCP affects conservation outcomes in terms of meeting wildlife species objectives. We then assess how these protection and restoration priorities change when integrated into an existing conservation plan called the Sagebrush Conservation Design (SCD; Doherty et al. 2022). We thus demonstrate how SCP can be used to create and expand adaptable decision‐support tools that accommodate newly available data resources and present new approaches to solving global conservation planning challenges that are transferable across a wide array of systems and species.

## 2 Study Area

**FIGURE 1:**
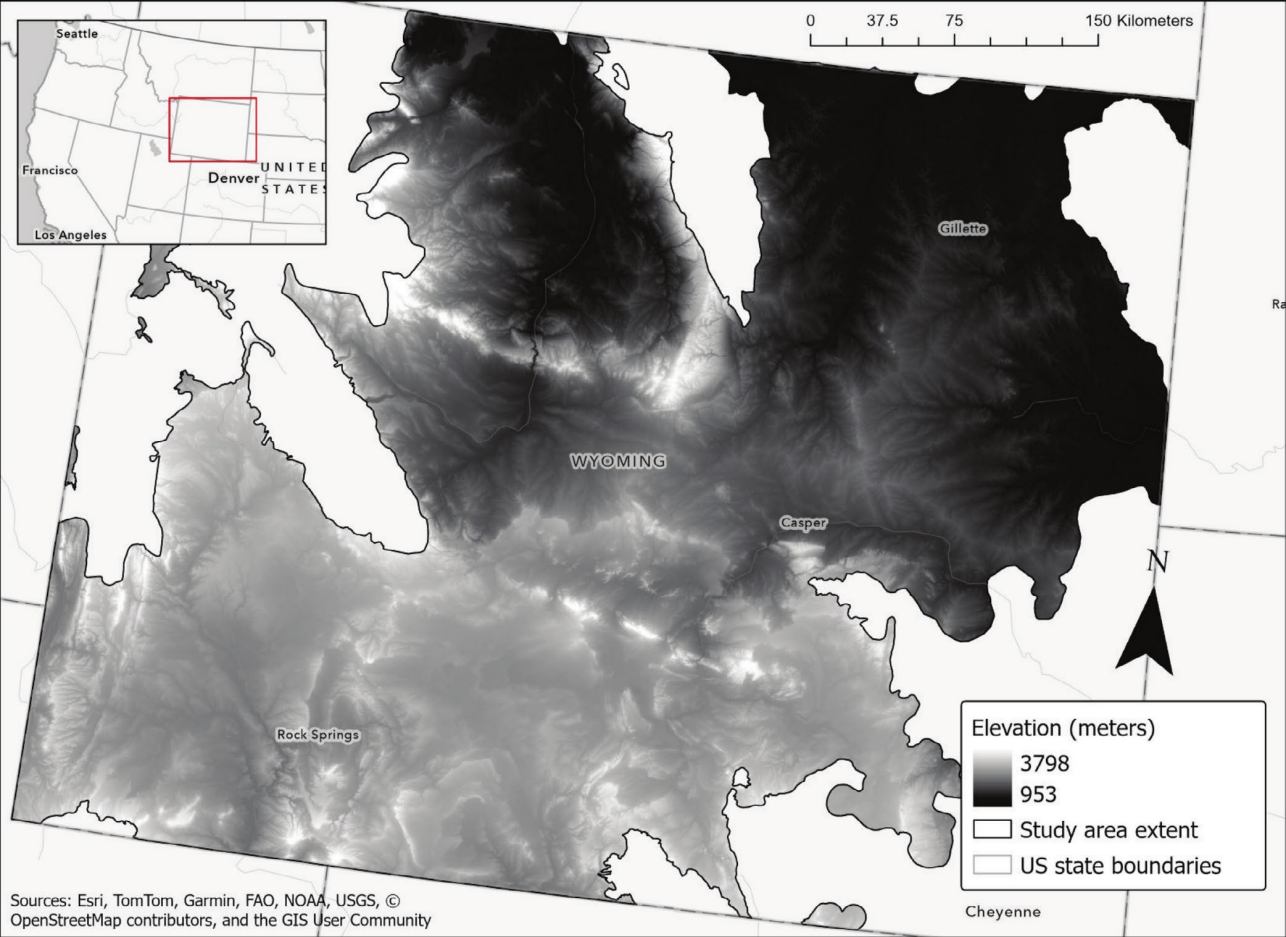
Map of the study area in which we considered sagebrush habitats for optimization problems, delineated by the current extent of the sagebrush biome in the state of Wyoming. Elevation gradient included for reference.

Our study area approximates the extent of sagebrush ecosystems within Wyoming, USA, an area covering 174,131 km^2^ (68% of the state) as delineated by O'Donnell et al. (2019) (Figure 1), with elevations between 953 and 3798 m (U.S. Geological Survey Earth Resources Observation and Science 2003). These semi‐arid ecosystems experience harsh winters with relatively short summers and heterogeneous precipitation patterns. Overstory vegetation is dominated by Wyoming big sagebrush ( *A. tridentata ssp. wyomingensis* ), with occasional salt flats supporting greasewood ( *Sarcobatus vermiculatus* ), saltbush (*Atriplex* spp.), and yellow rabbitbrush ( *Chrysothamnus viscidiflorus* ). Understory vegetation largely consists of forbs and bunchgrasses such as ricegrass ( *Achnatherum contractum* , *A. hymenoides* ) and bluebunch wheatgrass (*Pseudoroegnaria spicata*; Manier et al. 2011). The area serves as key habitat for both sagebrush‐obligate and ‐associated fauna (Davies et al. 2011; Van Lanen et al. 2023b), such as the greater sage‐grouse, sage thrasher ( *Oreoscoptes montanus* ), Brewer's sparrow ( *Spizella breweri* ), sagebrush sparrow ( *Artemisiospiza nevadensis* ), green‐tailed towhee ( *Pipilo chlorurus* ), and pygmy rabbit ( *Brachylagus idahoensis* ). The state's sagebrush biome also represents important habitat for migratory big game (Kauffman et al. 2020), including mule deer ( *Odocoileus hemionus* ), pronghorn ( *Antilocapra americana* ), and elk ( *Cervus elaphus* ).

Wyoming hosts some of the most contiguous sagebrush ecosystems remaining in the western United States and is considered a stronghold for the high‐profile greater sage‐grouse (Miller et al. 2012; Wisdom et al. 2012). Sagebrush management in the state is heavily focused on the species and guided by spatial resources, including occupied lek (i.e., breeding) sites and Wyoming's greater sage‐grouse core areas (hereafter, Wyoming core areas; Wyoming Governor's Sage‐Grouse Implementation Team 2015). The Wyoming core areas represent areas where greater sage‐grouse and their habitats would be most effectively conserved and where some land uses and activities are restricted to avoid negative effects to the species (Gordon 2019). Wyoming's sagebrush biome is administered by a mosaic of entities (Bureau of Land Management 2017) including private landowners (46%), the Bureau of Land Management (39%), state of Wyoming (7%), and other ownership types (including Tribal Lands; 8%). Oil and gas development is one of the prominent land uses in the area, with over 27,000 producing oil and gas wells in 2022 (Wyoming Oil and Gas Conservation Commission 2023). Additional land uses include surface mining (primarily coal), livestock grazing, and wind energy development (over 1300 turbines as of 2022; Hoen et al. 2018).

## 3 Methods

The initial iteration of PReSET (Duchardt et al. 2021) used the *prioritizr* package (Hanson et al. 2021) in Program R (R Core Team 2021) and a Gurobi optimizer (Gurobi Optimization and LLC 2021) to solve multi‐species restoration planning problems while considering varying spatial extents within southwestern Wyoming. *Prioritizr* uses mixed integer linear programming (MILP), which outperforms simulated annealing (used by popular software such as Marxan) in terms of faster processing time and generating results closer to optimality (Beyer et al. 2016; Schuster et al. 2020). Among MILP tools, *prioritizr* provides more flexibility to customize the use of diverse objectives, constraints, and penalties in the design of optimization problems (Hanson et al. 2021; Giakoumi et al. 2025), with Gurobi as the recommended optimizer (Hanson et al. 2021). The initial version of PReSET used presence/absence data to represent focal species, in combination with a single cost layer (sagebrush recovery time), to identify optimal sites for restoration action only. We built upon this by expanding the tool's capabilities to accommodate multiple conservation pathways, including options to explore sagebrush habitat restoration, protection, or both, for an integrated approach. This led to the development of a suite of problem sets that identify optimal sites for (1) protection of existing, intact sagebrush, (2) restoration of currently degraded sagebrush, and (3) a synthesis of protection and restoration actions within the context of an existing regional conservation plan (the SCD; Doherty et al. 2022). We also improved upon the original PReSET by expanding its spatial extent to cover the full sagebrush biome within the state of Wyoming, incorporating newly available species data (e.g., density estimates), and expanding the problem set portfolio to consider future climate conditions, habitat connectivity, and data uncertainty.

Our optimization tool is designed to be flexible with regard to geospatial input data, with input layers separated into three main categories: (1) *planning units* determine the pool of potential management sites (30‐m × 30‐m pixels) from which an optimal set of sites is selected, (2) *cost layers* represent some ecological or management constraint (monetary or non‐monetary) that we seek to minimize through selection of planning units, and (3) *feature layers* represent density or other quantifiable measures of potential habitat value for key species of conservation concern or interest, by which conservation targets can be defined and achieved. Below, we describe these input layers and how they inform our prioritizations in greater detail. We also discuss the structure and parameters of each optimization problem and analysis we conducted to investigate trade‐offs and synergies among the problems. All input layers were originally developed as 30‐m resolution raster layers or resampled to 30‐m resolution as described below.

### 3.1 Planning Units (Potential Management Sites)

#### 3.1.1 Degraded Sagebrush

We used sagebrush cover estimates (Rangeland Condition Monitoring Assessment and Projection (RCMAP) v3; Rigge et al. 2022) to delineate potentially suitable sagebrush sites (i.e., 30‐m resolution planning units) for restoring (i.e., planting or seeding) sagebrush where its cover had declined over time. We identified restoration planning units, representing degraded sagebrush sites, as pixels where sagebrush loss was at least two standard deviations above the mean change from historic cover (1985–1986) or the ecological potential cover (i.e., expected cover under undisturbed conditions; Rigge, Meyer, and Bunde 2021), or where both divergence from historic and potential cover were between one and two standard deviations above the mean (refer to Data S1 for further details, including Figures S1 and S2). Comparison with ecological potential cover was included to avoid missed opportunities to restore sites degraded before 1985.

#### 3.1.2 Intact Sagebrush

Similarly, we used sagebrush cover estimates (RCMAP v3; Rigge et al. 2022) to identify planning units representing intact sagebrush sites for habitat protection action. First, we excluded sites identified as degraded (described above) and then limited remaining sites to 30‐m pixels with ≥ 15% sagebrush cover (2019–2020; indicated by Connelly et al. 2000 as the minimum sagebrush cover for use by sage‐grouse). Both planning unit layers are publicly available in the associated ScienceBase data release (Shyvers et al. 2025; https://doi.org/10.5066/P14TYNTY).

### 3.2 Cost Layers (Ecological Constraints)

#### 3.2.1 Spring Soil Moisture Availability

We used a dataset of continuous values describing spring soil moisture availability across the sagebrush biome (based on climate averages from 1981 to 2010; O'Donnell and Manier 2022a) as an indicator of potential sagebrush restoration feasibility. Restoration of sagebrush ecosystems is challenging, and success is uncertain (Svejcar et al. 2017; Shriver et al. 2018), making it critical to guide restoration activities to where they are more likely to succeed. Moisture availability is an important predictor of sagebrush resilience and planting success within semi‐arid and arid shrub‐steppe (Minnick and Alward 2012; Chambers et al. 2014; Tarbox et al. 2024), and these soil climate data are moderately correlated with sagebrush cover (*R* ^2^ = 0.51; O'Donnell and Manier 2022b). For a measure of uncertainty, we calculated the coefficient of variation (CV) for this layer by dividing the spring soil moisture standard deviation layer by the layer of mean spring soil moisture for each pixel.

#### 3.2.2 Sagebrush Connectivity

We developed a data layer representing contemporary (2020) structural sagebrush connectivity across the sagebrush biome in Wyoming using an omnidirectional circuit‐based approach (McRae et al. 2008, 2016; Landau et al. 2021; Buchholtz et al. 2023) to model connections among pixels of sagebrush (hereafter, “sagebrush connectivity”). This approach uses electrical current as a proxy for random‐walk movement within a moving window (McRae et al. 2008) and expresses a measure of connectivity that has been widely used in wildlife and landscape ecology (Dickson et al. 2019). Key inputs for this model include the conductance surface, source layer, and the size of the moving window (30 km; this extent directly relates to the upper limit for an annual sage‐grouse home range (2975 km^2^; Connelly et al. 2000; Connelly et al. 2004), which has been linked to habitat changes leading to sage‐grouse extirpations (Aldridge et al. 2008)). We used the RCMAP v3 sagebrush fractional component for the conductance surface (Rigge et al. 2022) and set the threshold for identifying sources at 15% sagebrush cover. This allowed us to map potential connections among pixels with moderate sagebrush cover that matched our working definition of “intact” sagebrush. This layer is publicly available in the associated ScienceBase data release (https://doi.org/10.5066/P14TYNTY). Refer to Data S1 (including Figures S3–S5) for detailed methods describing how we derived these layers.

#### 3.2.3 Lek Connectivity (2020)

Connectivity of sagebrush habitat between leks reduces the risk of extirpation (Aldridge et al. 2008), increases lek persistence (Wann et al. 2023), and increases gene flow between leks and populations (Row et al. 2018; Zimmerman et al. 2022). To consider potential connectivity of greater sage‐grouse habitat among leks, we developed a connectivity map using contemporary (2020) sagebrush cover for the conductance surface (Rigge et al. 2022) and used contemporary (2010–2019) occupied lek locations as the source layer (O'Donnell et al. 2021). We weighted all leks equally (equal source strengths in Omniscape) so that outputs represented the potential connectivity among leks based on contemporary sagebrush cover between them. In Wyoming core areas, occupied leks are protected within a 0.6‐mile buffer (Gordon 2019). Because connectivity values were extremely high in the immediate area surrounding leks, we assumed that sites within state and federally defined lek protection buffers (which vary somewhat across states) already receive sufficient protections and masked them out of prioritization solutions by reclassifying all output raster cells within a 1‐km buffer of lek locations to zero values. The final map (hereafter, “lek connectivity”) represented the mean values over the 30‐km moving window. This layer is not available owing to data‐sharing restrictions related to the sensitivity of lek locations. For more information or to seek data access through a data‐sharing agreement, please contact the Wyoming Game and Fish Department (wgf.inforequest@wyo.gov).

#### 3.2.4 Lek Connectivity Loss (1985–2020)

In addition to prioritizing protection of existing connectivity, we created a map of greater sage‐grouse lek connectivity loss since 1985 to assess sites for opportunities to restore lost connectivity. We used the same methods as for the contemporary (2020) layer but with historical (1985) sagebrush cover as the conductance surface (Rigge et al. 2022). We then calculated the change in potential sagebrush connectivity among leks by subtracting the contemporary (2020) and historical (1985) lek connectivity output surfaces. Because increases in connectivity values over time can result from the redirection of current in the models, rather than representing actual improvements in habitat condition, we reclassified all positive values in this change layer to zero, thereby only reflecting predicted loss (negative change values) in connectivity between 1985 and 2020. This layer is publicly available in the associated ScienceBase data release (https://doi.org/10.5066/P14TYNTY).

#### 3.2.5 Future Sagebrush Projections

We used a dataset projecting future sagebrush percent cover (out to the 2080s) across our study area given a representative concentration pathway (RCP) emissions scenario of 4.5 (Rigge 2022). These layers were developed using fractional component cover data for rangeland functional groups, weather data from the 1985 to 2018 reference period, and soils and topography data to develop empirical models describing the spatiotemporal variation in component cover (Rigge 2022). RCP 4.5 is an intermediate emissions scenario based on the assumption that a range of technologies and strategies for reducing greenhouse gases will be effectively employed (IPCC 2014).

### 3.3 Feature Layers (Focal Species)

#### 3.3.1 Sagebrush‐Obligate and Sagebrush‐Associated Songbird Median Densities

We used a dataset representing median predicted songbird density (birds/km^2^) for four songbird species associated with sagebrush or shrub‐steppe ecosystems: Brewer's sparrow, sagebrush sparrow, sage thrasher, and green‐tailed towhee (Van Lanen et al. 2023b). These species are of moderate conservation concern within portions of their range (Partners in Flight 2023) and are of particular interest to land managers and conservationists. These predicted density layers were developed using density‐habitat relationships modeled in a hierarchical Bayesian framework and resource conditions spanning 2008 to 2020 (refer to Data S1 for further details; Van Lanen et al. 2023a; Van Lanen et al. 2023b). We also used the coefficient of variation (CV), a measure of variation in data relative to the mean, as a standardized measure of uncertainty across these and other data sets.

#### 3.3.2 Pygmy Rabbit Presence Probability

We used a dataset developed by Smith et al. (2019) representing the probability of pygmy rabbit presence, predicted across the species' geographic range. This predictive surface was developed using an ensemble of species distribution models from the program Maxent (Phillips et al. 2017) based on 10,420 pygmy rabbit occurrence records (from 2000 to 2019) and models of varying complexity, incorporating topographic, vegetation, fire, climate, and soil information (Smith et al. 2019).

#### 3.3.3 Greater Sage‐Grouse Mean Lek Abundance

We used contemporary (2019) mean estimates of greater sage‐grouse abundance at leks based on trends modeled from annual lek counts conducted between 1993 and 2019 and associated CV values (refer to Data S1 for details). These estimates were generated using a Bayesian state‐space model to estimate detectability from repeated lek counts each year (e.g., McCaffery et al. 2016; Monroe et al. 2022). Estimates were averaged across leks within population cluster polygons that represent ecologically significant management areas in Wyoming (“level 1” scale clusters; O'Donnell et al. 2019). To obtain a 30‐m raster matching the resolution of our other data inputs, we converted these polygons so that each 30‐m pixel value was equal to the average predicted value at the much larger “level 1” polygon extent. We used this layer to identify priority sites for restoration action because other feature layers used in restoration problems were also derived from “level 1” population clusters (refer to ‘Problem set #2’ below). This layer is publicly available in the associated ScienceBase data release (https://doi.org/10.5066/P14TYNTY).

#### 3.3.4 Greater Sage‐Grouse Lek Persistence Probability

Because the estimates of greater sage‐grouse abundance (above) were derived from “level 1” population cluster polygons rather than the 30‐m resolution of the other feature layers, we also used a map of greater sage‐grouse lek persistence probability (Wann et al. 2023) for optimization problems identifying priority sites for protection. This layer represents the probability that a hypothetical lek at each raster cell would remain active (i.e., have two or more male sage‐grouse lekking at the site) across two periods: 2000–2009 and 2010–2019. It was modeled across the range at 30‐m resolution and based on contemporary landscape characteristics of habitats up to 15 km away (e.g., sagebrush cover, pinyon‐juniper cover, topography, precipitation, disturbance, and landscape configuration metrics; Wann et al. 2023). To account for regionally varying sage‐grouse habitat associations, the slopes of habitat covariates were allowed to vary within second‐order scale clusters (Stiver et al. 2010).

### 3.4 Optimization Problem Design

We standardized planning unit, cost, and feature layers in ArcGIS Pro (v.2.9.2 ESRI Inc. 2021) by snapping to a common layer, ensuring all cells aligned correctly (spring soil moisture availability, greater sage‐grouse lek persistence probability, and pygmy rabbit presence probability layers only), and clipping each layer to our study area extent. We rescaled all feature layer values between 0 and 1 to aid optimization computations. We then used *prioritizr*, with the Gurobi solver, to develop SCP problems using the following settings:

1. Minimum‐set objectives to find solutions that minimize the total overall cost layer value across selected pixels
2. Relative species targets = 0.2 (i.e., protecting or restoring a minimum of 20% of each feature layer's values)
3. Binary decisions where each available pixel can either be selected [1] or not selected [0] in a solution
4. Gap allowance = 0.01 (i.e., the deviation from targets allowed to reduce runtimes)

For some restoration problems, we utilized boundary penalties to encourage clustering of selected pixels (Hanson et al. 2021) around existing, intact sagebrush habitat or into large, contiguous restoration sites to facilitate restoration success and management efficiency. We calibrated these penalties for each problem in which they were used (refer to details in Table 1 and Data S1) to ensure their influence was aligned with problem objectives and context (Ardron et al. 2010) and employed an edge factor = 0.5 to avoid overly penalizing planning units along study area boundaries (Hanson et al. 2021). We also sometimes used nominal boundary penalties (0.00001; refer to Table 1) to facilitate model performance by preventing spatial fragmentation of prioritization problems with low constraints (Hanson 2021). For each problem, we also calculated summary statistics (i.e., median, first and third quartiles, minimum, and maximum) of cost and feature layer values of selected sites. Additionally, we calculated summary statistics across all intact sagebrush, degraded sagebrush, and other conservation areas (e.g., Wyoming core areas; Data S2, Table S1). We describe the methods and design for each problem set below and summarize their setup and spatial data inputs for side‐by‐side comparison in Table 1.

**TABLE 1:** List of optimization problem structures and inputs used to prioritize sites for conservation action (protection and/or restoration) within sagebrush ecosystems of Wyoming, USA.

Problem	Planning units	Mask(s)	Cost layer	Feature layers	Locked‐in features	Boundary penalties (edge factor = 0.5)	Uncertainty layers
#1: Identifying priority protection sites							
1a (no cost): Protect sagebrush species' habitats	Intact sagebrush	—	Null (all values = 1)	Sagebrush‐obligate and ‐associated songbird mean densities, greater sage‐grouse lek persistence probability, pygmy rabbit presence probability	—	0.00001 (*all species* only)	—
1b (connectivity): Protect sagebrush species' habitats with high sagebrush connectivity	Intact sagebrush	—	Sagebrush connectivity (transposed values)	Sagebrush‐obligate and ‐associated songbird mean densities, greater sage‐grouse lek persistence probability, pygmy rabbit presence probability	—	—	—
1c (future sagebrush): Protect sagebrush species' habitats with high predicted future sagebrush cover	Intact sagebrush	—	Future sagebrush projections (transposed values)	Sagebrush‐obligate and ‐associated songbird mean densities, greater sage‐grouse lek persistence probability, pygmy rabbit presence probability	—	—	—
1d (low resilience): Protect sagebrush species' habitats with low resilience	Intact sagebrush	None, ≥ 5%, ≥ 10%, and ≥ 15% projected sagebrush cover in 2080 (Rigge 2022)	Spring soil moisture availability	Sagebrush‐obligate and ‐associated songbird mean densities, greater sage‐grouse lek persistence probability, pygmy rabbit presence probability	—	0.00001	—
#2: Identifying priority restoration sites							
2a (high resilience): Maximize restoration success	Degraded sagebrush	—	Spring soil moisture availability (transposed values)	Ecological potentials^a^ (sagebrush‐obligate and ‐associated songbirds, greater sage‐grouse mean lek abundance, pygmy rabbit)	Intact sagebrush sites	0.006 to 0.024 (individual species), 0.1 (*all species*)	—
2b (uncertainty): Assess trade‐offs relating to data uncertainty	Degraded sagebrush	—	Spring soil moisture availability (transposed values)	Ecological potentials^a^ (sagebrush‐obligate and ‐associated songbirds, greater sage‐grouse mean lek abundance)	—	—	Coefficient of variation (spring soil moisture availability, sagebrush‐obligate and ‐associated songbirds, greater sage‐grouse mean lek abundance)
#3: Enhancing existing conservation strategies							
3a (SCD protection): Connectivity‐focused protection within SCD core sagebrush and growth opportunity areas	Intact sagebrush	Core habitats and growth opportunity areas (Doherty et al. 2022)	Lek connectivity (2020; transposed values)	Sagebrush‐obligate and ‐associated songbird mean densities, greater sage‐grouse lek persistence probability, pygmy rabbit presence probability	—	—	—
3b (SCD resilience): Resilience‐focused restoration within SCD core sagebrush and growth opportunity areas	Degraded sagebrush	Core habitats and growth opportunity areas (Doherty et al. 2022)	Spring soil moisture availability (transposed values)	Ecological potentials^a^ (sagebrush‐obligate and ‐associated songbirds, greater sage‐grouse mean lek abundance, pygmy rabbit)	Priority conservation sites from 3a	2.5	—
3c (SCD connectivity): Restore areas of connectivity loss within SCD core sagebrush and growth opportunity areas	Degraded sagebrush	Core habitats and growth opportunity areas (Doherty et al. 2022)	Lek connectivity loss (1985 to 2020)	Ecological potentials^a^ (sagebrush‐obligate and ‐associated songbirds, greater sage‐grouse mean lek abundance, pygmy rabbit)	Priority conservation sites from 3a	1.5	—

*Note:* Planning units represent the pool of potential management sites (30 × 30 m pixels) from which an optimal set of priority sites is selected; masks are used to refine or narrow the set of planning units; cost layers represent constraints or penalties to be minimized through selection of planning units; feature layers represent density or other population metrics for focal species of conservation concern; locked‐in features represent a subset of the planning units that are automatically selected in the optimization solutions; boundary penalties represent a uniquely calibrated scalar value applied to cluster selected planning units in optimization solutions; and uncertainty layers represent coefficient of variation values associated with cost or feature layers. All problems used global relative targets = 0.20 for feature input values, representing protection or restoration of at least 20% of total density for a focal species, binary decisions (0,1), a boundary penalty (with edge factor = 0.5), and a gap allowance = 0.01.

^a^ Values representing the ecological potential of restoring degraded sagebrush sites. These were calculated by averaging focal species' feature layer values (e.g., songbird median densities) for all intact sagebrush sites across each level 1 cluster polygon (O'Donnell et al. 2019), representing ecologically significant management areas, and applying that average to all degraded sagebrush sites within the same polygon. SCD refers to the Sagebrush Conservation Design strategy (Doherty et al. 2022).

### 3.5 Problem Set #1: Identifying Priority Protection Sites

We designed our first set of problems to protect existing, intact sagebrush habitats for multiple species from future development or degradation. We used intact sagebrush to represent the planning units for each variation of this problem. Each problem in this set used all four songbird species' density estimates, greater sage‐grouse lek persistence probability, and pygmy rabbit presence probability as feature layer inputs. We allowed these problems to differ in their cost layers (i.e., sagebrush connectivity, future sagebrush projections, and spring soil moisture availability) to evaluate how different management priorities may influence conservation delivery.

#### 3.5.1 1a (No Cost): Protect Sagebrush Species' Habitats

We developed a baseline protection problem to identify the most important protection sites, regardless of connectivity, restoration feasibility, or climate change projection constraints, by using a uniform cost layer input (all values = 1). We ran seven iterations of this problem, adjusting the relative targets to consider all species together (i.e., all feature layer targets = 0.2) and each species individually (e.g., Brewer's sparrow target = 0.2 and all other species' targets = 0.0) to investigate differences in priority protection areas based on varying habitat needs of different focal species. In contrast to the boundary penalties we used for restoration problems, here we applied a considerably smaller boundary penalty (0.00001) to the all‐species iteration as an additional constraint to avoid spatial fragmentation of prioritized sites (Hanson 2021). To investigate support for the greater sage‐grouse umbrella species concept in Wyoming (Rowland et al. 2006; Aldridge et al. 2024), we calculated the proportion of total species' feature layer values within selected pixels. Additionally, we calculated the proportion of species' feature layer values across all intact sagebrush planning units (i.e., selected and unselected) within the Wyoming core areas and SCD core sagebrush areas (CSA).

#### 3.5.2 1b (Connectivity): Protect Sagebrush Species' Habitats With High Sagebrush Connectivity

To prioritize habitat connectivity while identifying important sites for protection for multiple species, we replaced the uniform (all values = 1) cost layer from the all‐species iteration of the baseline problem (1a, no cost) with the contemporary sagebrush connectivity layer. This provided sufficient constraint such that the nominal boundary penalty was not needed to minimize spatial fragmentation of selected sites. Because minimum‐set problems prioritize minimizing the values of the cost layer in selected planning units, we modified this layer to preferentially select areas with high connectivity of sagebrush habitats by transposing the values to be ≥ 1 using Equation (1):

(1) $$-1\times \text{raster value}+\left({\text{raster value}}_{\max}+1\right)$$

#### 3.5.3 1c (Future Sagebrush): Protect Sagebrush Species' Habitats With High Predicted Future Sagebrush Cover

A changing climate may render some sites unsuitable for restoring sagebrush in the future. To investigate how climate change might influence strategic planning for the conservation of habitats to benefit multiple species, we used the all‐species baseline problem without a boundary penalty (as in 1c, connectivity); this time replacing the cost layer with future sagebrush projections (again transforming the values using Equation 1). This allowed us to prioritize the selection of sagebrush species' habitats that are expected to retain sagebrush cover into the latter part of the century.

#### 3.5.4 1d (Low Resilience): Protect Sagebrush Species' Habitats With Low Resilience

To emphasize protection of intact sagebrush sites that may be difficult or impossible to recover if degraded, we again used the all‐species baseline problem, this time substituting spring soil moisture as our cost layer. Additionally, because sagebrush in low‐resilience sites may be unlikely to persist, even if protected from development or degradation, we applied masks of projected future sagebrush cover to our intact sagebrush planning unit layer. We ran four iterations of this problem where we masked the planning unit layer using four thresholds of future sagebrush cover: no threshold and cover values ≥ 5%, 10%, and 15%. As with problem 1a (no cost), we applied a small boundary penalty (0.00001) to prevent spatial fragmentation (Hanson 2021).

### 3.6 Problem Set #2: Identifying Priority Restoration Sites

Our second set of problems was designed to identify priority areas for sagebrush restoration. We prioritized sites where restoration is most likely to be successful (based on spring soil moisture availability) and create valuable habitat for focal species. Prioritizing site‐scale restoration is challenging because degraded sites, by definition, exhibit lower abundance estimates for the focal species that drive prioritization algorithms. Estimates of current abundance (or other species values) are therefore uninformative for identifying opportunities to restore wildlife habitat, creating a need for projected changes in species abundance in response to sagebrush restoration. We generated estimates of potential habitat value (i.e., “ecological potentials”) for pygmy rabbit and songbird species by aggregating species metrics across 2020 locations with intact sagebrush and applying them to each degraded sagebrush planning unit. To develop these estimates, we first averaged values for each of these focal species, overlapping intact sagebrush within level 1 management cluster polygons (O'Donnell et al. 2019), using the zonal statistics tool in ArcGIS Pro, then applied those values to all degraded sagebrush sites within the same polygon. For both problems in this set, we used the potential habitat value layers generated from this process for each songbird species and pygmy rabbit as the feature layer inputs and the degraded sagebrush layer to define planning units. For greater sage‐grouse, we used the lek abundance layer, which was originally generated at the scale of level 1 clusters and thus did not require the processing described above.

#### 3.6.1 2a (High Resilience): Maximize Restoration Success

To prioritize sites that maximize the potential for successful sagebrush restoration and minimize expected recovery times, we used spring soil moisture as the cost layer input after transforming the layer using Equation (1). To promote the selection of priority restoration sites surrounding intact sagebrush, we then created a planning unit layer by merging the degraded sagebrush and intact sagebrush layers. We applied the intact layer as a locked‐in feature (a function ensuring its automatic inclusion as a selected unit) and applied boundary penalties uniquely calibrated for each iteration (refer to Data S1 for details; Ardron et al. 2010; Hanson et al. 2021). As in problem 1a (no cost), we ran several iterations of this problem to investigate differences in solutions aimed at prioritizing management for all species compared to individual species.

#### 3.6.2 2b (Uncertainty): Assess Trade‐Offs Related to Data Uncertainty

We explored the potential for uncertainty in our spatial data inputs to address variability in risk tolerance during decision‐making processes. We used the same base‐problem setup as in problem 2a (high resilience) but excluded pygmy rabbit presence probability because no uncertainty data were available for that feature layer. We assessed uncertainty by applying weighted penalties, based on associated CV values, to both the cost and feature layer inputs. We incrementally increased those penalty weights across prioritization iterations, using Equations (2) and (3) to calculate the weighted input layer (i.e., “utility” values):

(2) $${\text{Cost}}_{\text{utility}}={\text{Cost}}_{\text{value}}+\left(\text{Penalty}\_\text{weight}\times {\text{CostCV}}_{\text{value}}\right)$$

(3) $${\text{Feature}}_{\text{utility}}={\text{Feature}}_{\text{value}}-\left(\text{Penalty}\_\text{weight}\times {\text{FeatureCV}}_{\text{value}}\right)$$

We then reclassified any resulting negative raster values to zero. We ran nine iterations using penalty weights of 0.0 to 0.8, increasing by increments of 0.1, to represent increasing levels of risk avoidance among end‐users. For each iteration, we applied static targets for each feature layer based on a no‐penalty problem and applied these using the absolute targets option in *prioritizr*. We overlapped the resulting solutions for all iterations to identify areas or regions that are relatively more robust to uncertainty in the input layers. We did not use boundary penalties in this problem because unique penalty values would need to be calibrated for each iteration, effectively precluding direct comparison of differently weighted uncertainty solutions. Similarly, we also excluded the locked‐in “intact” sites used in 2a (high resilience) as this would prevent our ability to compare results.

### 3.7 Problem Set #3: Enhancing Existing Conservation Strategies

This set of problems was designed to incorporate elements from problem sets #1 (identifying priority protection sites) and #2 (identifying priority restoration sites) into an existing conservation strategy (the SCD; Doherty et al. 2022). The SCD delineates sagebrush habitats into broad‐scale categories based on vegetation cover and an index of human development to guide active, cross‐agency management focused on a “defend and grow the core” approach (Doherty et al. 2022). To aid managers in prioritizing the protection and restoration of sagebrush habitat within vast landscapes designated as growth opportunity areas (GOA) and CSA in the SCD, we used estimates of wildlife species' density/presence/persistence, lek connectivity, and sagebrush restoration potential. While centered on identifying priority sites for greater sage‐grouse, this problem also considered the needs of a diverse set of sagebrush‐obligate and ‐associated species of conservation concern to facilitate a multi‐species management approach.

#### 3.7.1 3a (SCD Protection): Connectivity‐Focused Protection Within SCD Core Sagebrush and Growth Opportunity Areas

This problem was modified from 1b (connectivity) with two key adjustments: we (1) masked the planning unit layer (i.e., our intact sagebrush layer) using the CSA and GOA raster layers (Doherty et al. 2022) to prioritize these SCD‐identified areas, and (2) substituted the sagebrush connectivity layer with our 2020 lek connectivity layer as the cost layer to focus efforts on areas with relatively high connectivity between occupied greater sage‐grouse leks. We again transposed the cost layer values using Equation (1) to prioritize the selection of sites with the greatest connectivity.

#### 3.7.2 3b (SCD Resilience): Resilience‐Focused Restoration Within SCD Core Sagebrush and Growth Opportunity Areas

To prioritize restoration among resilient sites within GOA and CSA areas identified in the SCD, we used the setup from problem 2a (high resilience), with three key adjustments. We (1) masked the degraded sagebrush layer in SCD areas (as in 3a, SCD protection) to further refine the planning units, (2) used the solution from problem 3a (i.e., selected priority protection sites) as a locked‐in layer to generate a restoration solution that promoted connectivity of restoration sites with priority protection sites, and (3) calibrated boundary penalties with edge factor = 0.5, again identifying the best scalar value (refer to Data S1 for details) to cluster selected restoration sites around the priority protection sites identified in 3a (Ardron et al. 2010; Hanson et al. 2021).

#### 3.7.3 3c (SCD Connectivity): Restore Areas of Connectivity Loss Within SCD Core Sagebrush and Growth Opportunity Areas

To prioritize restoration of sites within GOAs and CSAs where connectivity loss occurred over the past 30 years, we applied the setup from problem 3b (SCD resilience), except we used our lek connectivity loss (1985–2020) layer as the cost layer input to prioritize restoration to restore lost connectivity. To avoid optimization errors that would occur from the use of negative cost values, we transposed the connectivity loss layer so that all values would be ≥ 1 using Equation (4), where |Cost_min_| represents the absolute minimum value in the raster layer.

(4) $${\text{Cost}}_{\text{positive}}={\text{Cost}}_{\text{value}}+\left(\left|{\text{Cost}}_{\min}\right|+1\right)$$

## 4 Results

We generated binary (1 = selected, 0 = not selected) 30‐m raster solution layers for each problem, representing optimal sites for meeting relative species targets given specified constraints. These layers therefore optimize spatial allocation of management actions that could protect or restore 20% of each focal species' feature layer values (i.e., songbird densities, greater sage‐grouse lek persistence probability, pygmy rabbit presence probability) across all planning units. Below, we compare and contrast characteristics of sites selected in each problem, as well as sites identified in two existing conservation strategies that overlap our study area (Wyoming core areas and SCD GOAs and CSAs). Prioritization maps for each problem set are available via the publicly accessible ScienceBase data release (https://doi.org/10.5066/P14TYNTY).

### 4.1 Problem Set #1: Identifying Priority Protection Sites

**FIGURE 2:**
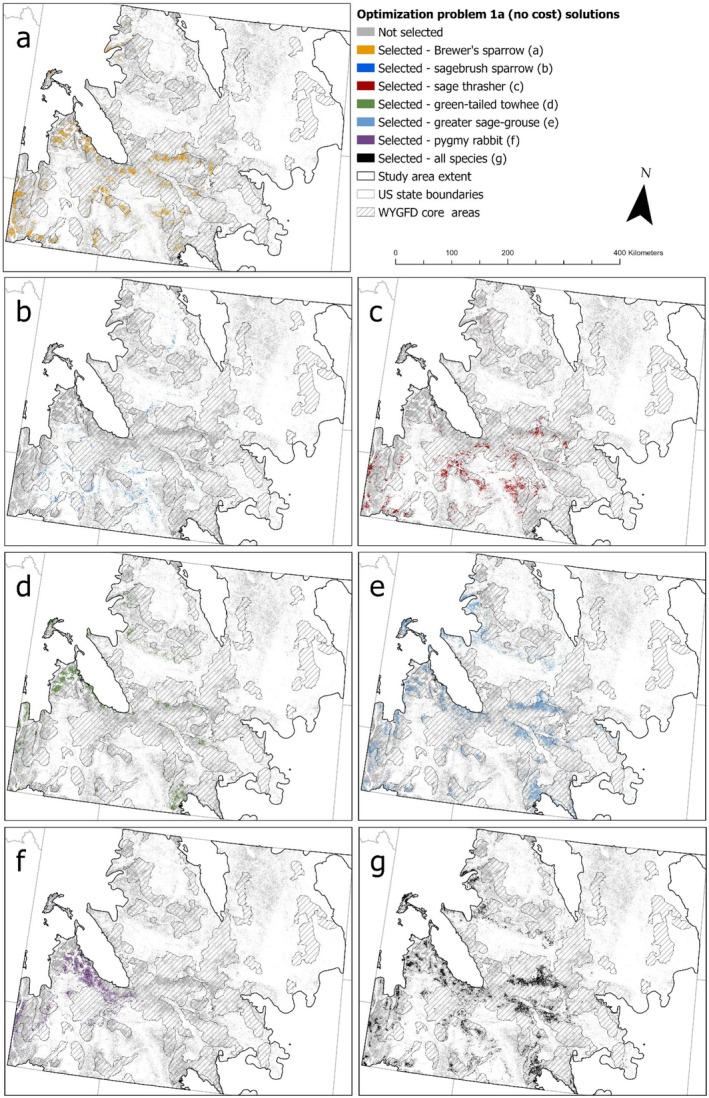
Solutions for optimization problem 1a (no cost), identifying priority protection sites in Wyoming's sagebrush steppe biome. Optimizations used contemporary species density, presence probability, or lek persistence probability for species of conservation concern to prioritize habitat protection for (a) Brewer's sparrow ( *Spizella breweri* ), (b) sagebrush sparrow ( *Artemisiospiza nevadensis* ), (c) sage thrasher ( *Oreoscoptes montanus* ), (d) green‐tailed towhee ( *Pipilo chlorurus* ), (e) greater sage‐grouse ( *Centrocercus urophasianus* ), (f) pygmy rabbit ( *Brachylagus idahoensis* ), and (g) all species combined. Wyoming's greater sage‐grouse core area polygons (Wyoming core areas) represent current focal management areas for greater sage‐grouse within the state (Wyoming Governor's Sage‐Grouse Implementation Team 2015).

In problem 1a (no cost), protecting 20% of sagebrush‐obligate and ‐associated species' populations within intact sagebrush habitats of Wyoming required an area that ranged from 81,774 ha (for sagebrush sparrow) to over half a million hectares (for greater sage‐grouse; Table 2). The area needed to meet protection targets for all species was similar to that for greater sage‐grouse only (Table 2), and selected sites showed considerable geographic overlap but with some important differences, particularly at lower elevations (Figure 2). Single‐species iterations of problem 1a (no cost) that focused on sagebrush sparrow and green‐tailed towhee protected the lowest proportions of other species' feature layer values, while the greater sage‐grouse iteration protected the highest proportions of other species' feature layer values (Table 2). The all‐species iteration of problem 1a (no cost) protected the minimum proportion of feature layer values possible to achieve protection targets (i.e., 0.20) for sagebrush sparrow and greater sage‐grouse, whereas outcomes for all other species exceeded minimum targets (ranging from 0.24 to 0.28; Table 2). Single‐species iterations of problem 1a (no cost) for Brewer's sparrow and sage thrasher selected sites supporting high densities and probabilities of most non‐target species, while the green‐tailed towhee and sagebrush sparrow iterations selected sites supporting the lowest densities and probabilities of non‐target species (Figure S1 in Data S2).

**TABLE 2:** Proportion of total species' feature layer values included within sites selected in problem set #1 and problem 2a (high resilience), as well as across all intact sagebrush planning units within Wyoming's core areas and Sagebrush Conservation Design (SCD) core sagebrush areas.

Problem	Area (ha)	Proportion of species' feature values within intact sagebrush protected under each problem					
		Greater sage‐grouse lek persistence probability	Pygmy rabbit presence probability	Brewer's sparrow density	Green‐tailed towhee density	Sagebrush sparrow density	Sage thrasher density
(1a) Greater sage‐grouse	548,359	0.20	0.23	0.23	0.31	0.10	0.20
(1a) Pygmy rabbit	271,780	0.09	0.20	0.14	0.10	0.16	0.15
(1a) Brewer's sparrow	294,645	0.10	0.16	0.20	0.16	0.10	0.21
(1a) Green‐tailed towhee	205,910	0.07	0.08	0.08	0.20	0.01	0.04
(1a) Sagebrush sparrow	81,774	0.03	0.04	0.03	0.01	0.20	0.05
(1a) Sage thrasher	214,717	0.07	0.12	0.13	0.07	0.17	0.20
(1a) All species	549,435	0.20	0.24	0.25	0.28	0.20	0.25
(1b) Connectivity	557,297	0.20	0.28	0.28	0.31	0.20	0.26
(1c) Future sagebrush	562,956	0.20	0.24	0.22	0.29	0.20	0.20
(1d) Low resilience (no mask)	601,444	0.20	0.26	0.25	0.20	0.35	0.30
(1d) Low resilience (≥ 5% future sagebrush cover)	586,180	0.20	0.27	0.25	0.20	0.34	0.31
(1d) Low resilience (≥ 10% future sagebrush cover)	572,828	0.20	0.28	0.24	0.23	0.20	0.25
Wyoming core areas	1,619,535	0.56	0.56	0.64	0.60	0.51	0.65
SCD core sagebrush areas	1,639,033	0.57	0.62	0.70	0.62	0.69	0.76
(2a) All species (high resilience)	194,147	0.06	0.06	0.06	0.08	0.06	0.05

*Note:* For single‐species iterations of problem 1a (no cost), protection targets were set to 0.20 for that species, and no targets were set for other species. For the remaining all‐species problems (1a (no cost) and 2a (high resilience)), each species' target was set to 0.20. For problem 2a (high resilience), proportional increases in each species' feature layer values are shown based on derived ecological potentials of species' feature layer values (refer to Section 3 for details). Cells where the 0.20 target was met exactly are indicated by the palest shade of green, with darkening shades of green representing increased proportions of feature values protected or restored for a species. Darker shades of purple indicate reduced protection or restoration.

The Wyoming core areas contained three times the amount of intact sagebrush as was selected by the all‐species iteration of problem 1a (no cost, Table 2). Since the objective of the all‐species problem was to identify sites that could protect 20% of each species' population, we compared our results to the protection provided by Wyoming core areas for each species based on a target of 60% (i.e., three times the 20% targets for intact sagebrush). The Wyoming core areas met or exceeded this target for Brewer's sparrow, green‐tailed towhee, and sage thrasher and were close to but lower than this target for the other species, particularly sagebrush sparrow (Table 2). However, estimated densities of green‐tailed towhee, sage thrasher, and Brewer's sparrow, as well as lek persistence probability of greater sage‐grouse, were lower in Wyoming core areas than in sites selected in problem 1a (no cost; Figure S2 in Data S2).

**FIGURE 3:**
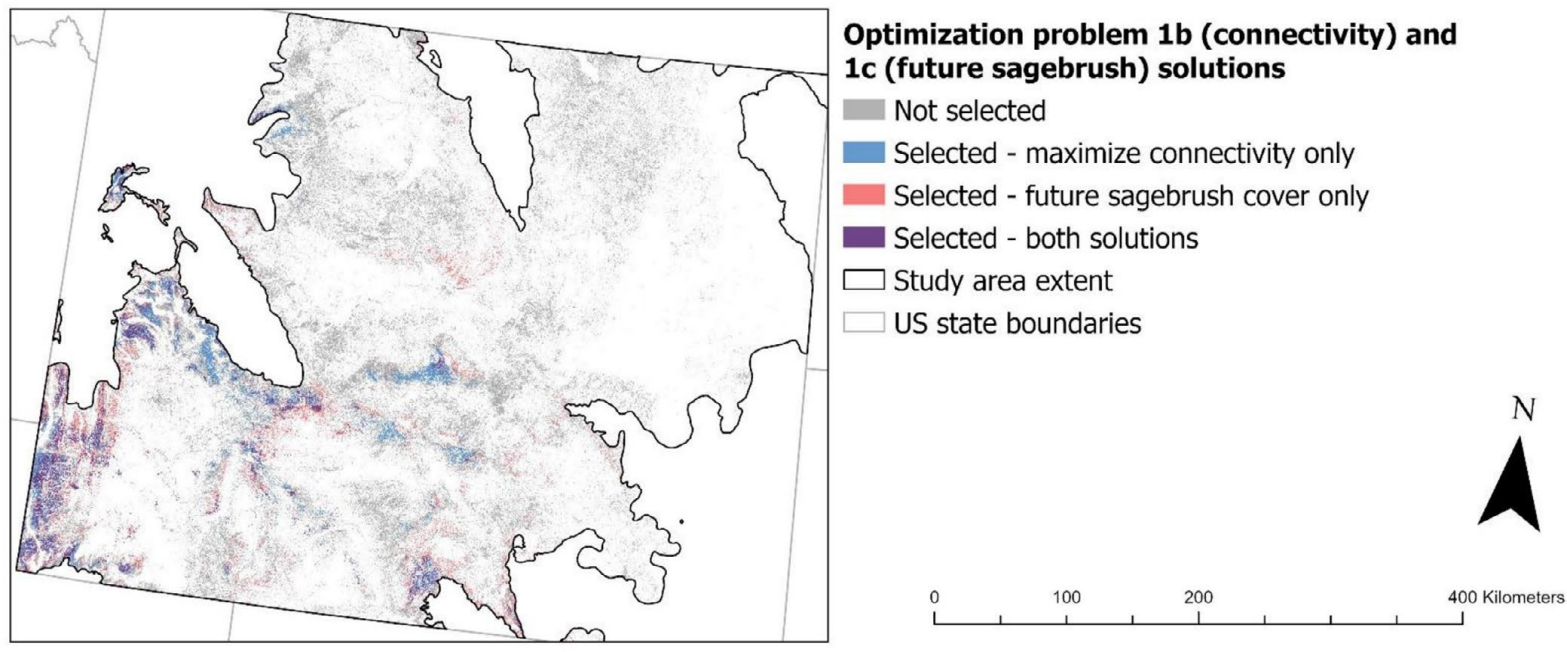
Solutions for optimization problems 1b (connectivity) and 1c (future sagebrush), identifying priority protection sites in Wyoming's sagebrush steppe biome. Optimization solutions prioritized habitat protection for focal species while maximizing connectivity of intact sagebrush sites (1b, blue) or predicted future sagebrush cover in the 2080s, given a representative concentration pathway (RCP) 4.5 climate scenario (1c, red; Rigge 2022).

**FIGURE 4:**
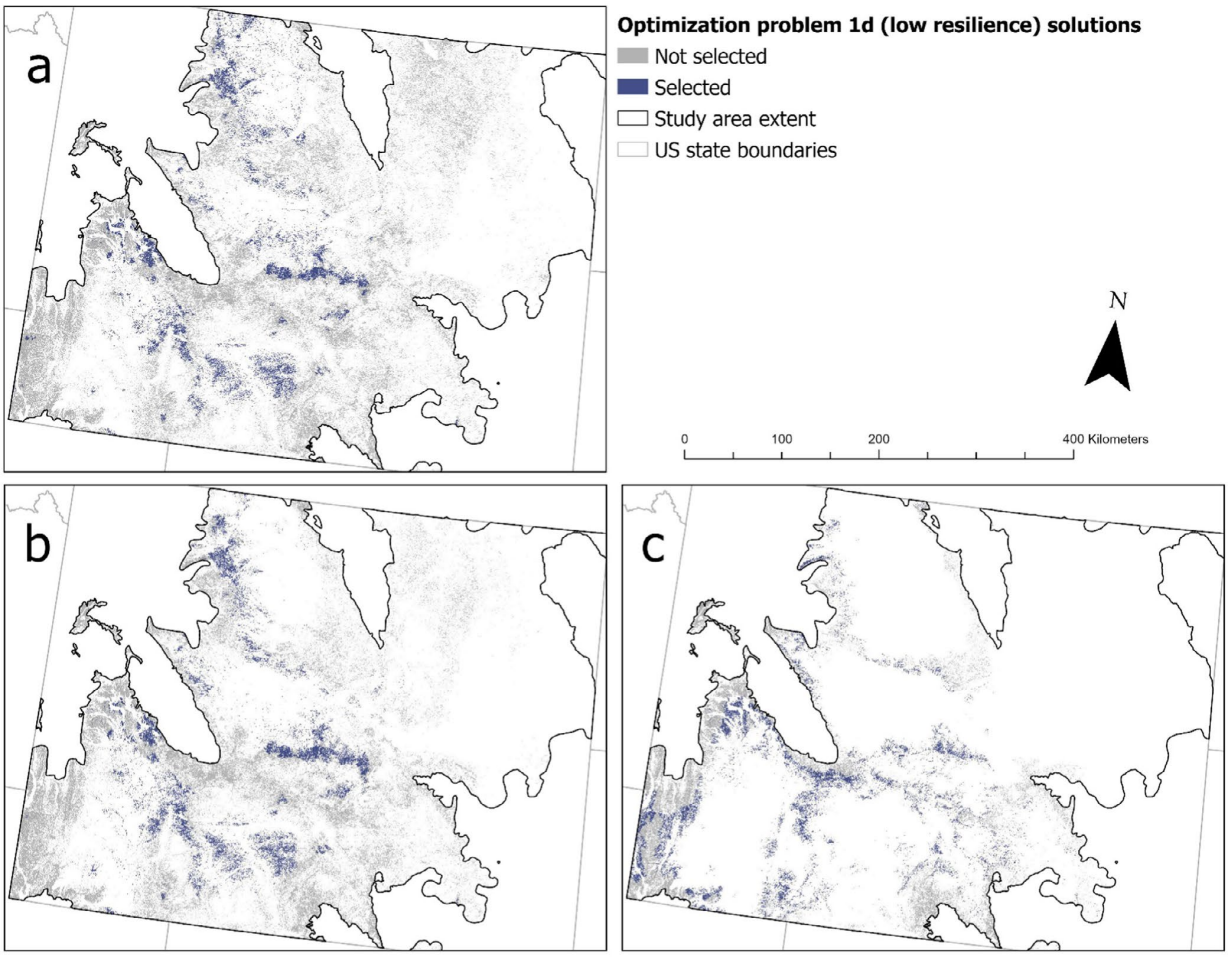
Solutions for optimization problem 1d (low resilience), identifying priority protection sites in Wyoming's sagebrush steppe biome. Optimization solutions prioritized habitat protection for focal species while focusing on sites predicted to be less resilient to disturbance or degradation based on spring soil moisture estimates (O'Donnell and Manier 2022a). We applied masks to define the pool of available sites for selection based on varying thresholds for percent sagebrush cover required to persist into the later part of the century (2080s, given a representative concentration pathway (RCP) 4.5 climate scenario; Rigge 2022). We applied future percent cover thresholds of 0% (i.e., no threshold) (panel a), ≥ 5% (panel b), ≥ 10% (panel c), and ≥ 15% (for which no feasible solution was found).

Adding cost layers to prioritize protection of sites with high sagebrush connectivity (problem 1b, connectivity) and which are expected to support future sagebrush (problem 1c, future sagebrush) to the all‐species protection optimization resulted in modest increases to the area required to meet protection targets. However, protecting sites with low ecological resilience (problem 1d, low resilience) required considerably more area than for other problems (Table 2). The degree of protection provided to focal species also varied between problems using different cost layers. Maximizing sagebrush connectivity (problem 1b, connectivity) provided greater protection for pygmy rabbit, Brewer's sparrow, and green‐tailed towhee than problem 1a (no cost), whereas maximizing future sagebrush cover (problem 1c, future sagebrush) provided less protection for Brewer's sparrow and sage thrasher (Table 2). Problem 1d (low resilience) provided greater protection for pygmy rabbit, sagebrush sparrow, and sage thrasher, but less for green‐tailed towhee (Table 2). However, masking planning units for problem 1d (low resilience) to areas expected to retain at least 10% sagebrush cover in the future resulted in less protection for sagebrush sparrow and sage thrasher, similar to problem 1a (no cost, Table 2). Feature layer values for each species (e.g., density, lek persistence probability) were generally similar between sites selected with no cost layer (1a) and sites selected using cost layers (1b–1d; Figure S2 in Data S2). However, problems 1c (future sagebrush) and 1d (low resilience) selected sites with lower values for some species, and all iterations of problem 1d selected sites with higher densities of sagebrush sparrow (Figure S2 in Data S2). Geographically, problems 1b (connectivity) and 1c (future sagebrush) selected many of the same sites as the all‐species iteration of problem 1a (no cost), although they also diverged in certain regions (Figure 3). Spring soil moisture availability and predicted future sagebrush cover within selected sites also varied considerably between problems, while lek connectivity was more consistent (Figure S3 in Data S2). Spring soil moisture availability was greater across sites selected under problems 1a (no cost), 1b (connectivity), and 1c (future sagebrush) than for all iterations of problem 1d (low resilience), while future sagebrush cover was greatest for problems 1b, 1c, and the iteration of problem 1d masked for 10% future sagebrush cover (Figure S3 in Data S2). Masking planning units of problem 1d (low resilience) to areas expected to retain at least 5% sagebrush cover in the future made little to no difference in cost layer values (Figure S3 in Data S2) or geographic distribution (Figure 4). Masking planning units for problem 1d (low resilience) to areas expected to retain at least 10% sagebrush cover in the future resulted in selected sites with greater spring soil moisture availability, future sagebrush cover, and elevation, and a markedly different geographic distribution compared to other iterations of problem 1d (Figure 4). Masking problem 1d (low resilience) to optimize protection among intact sagebrush expected to retain at least 15% sagebrush cover in the future was infeasible due to the lack of available sites projected to retain ≥ 15% sagebrush cover by the 2080s.

### 4.2 Problem Set #2: Identifying Priority Restoration Sites

**FIGURE 5:**
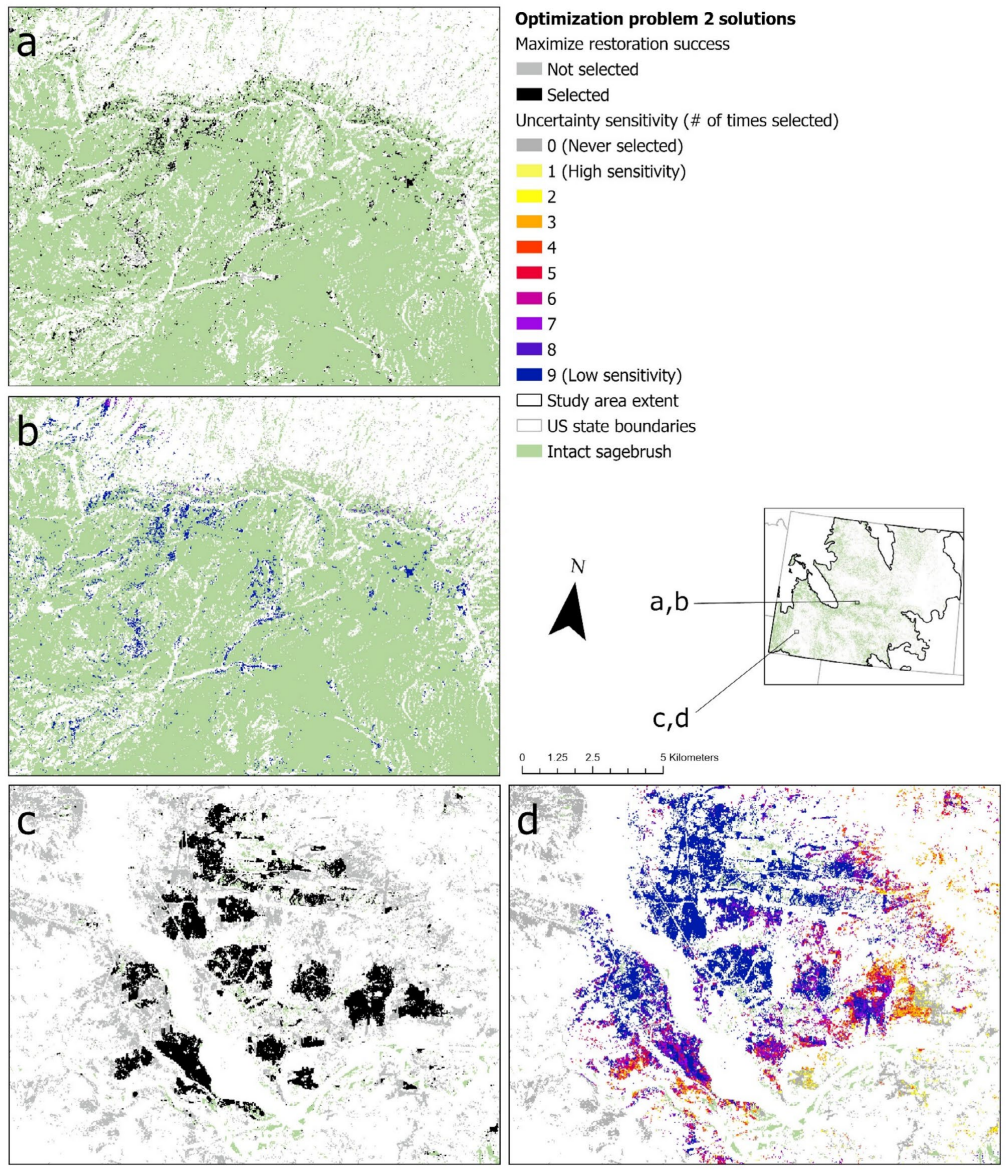
Solutions for optimization problem set #2, identifying priority restoration sites in Wyoming's sagebrush steppe biome. Optimizations used spring soil moisture availability (O'Donnell and Manier 2022a) to prioritize restoration where it was most likely to succeed and estimates of greater sage‐grouse lek abundance and potential habitat value for other focal species so that restoration efforts would benefit sagebrush‐obligate and ‐associated species. (Panel a) highlights an area where restoration is prioritized because it could augment large, contiguous patches of intact sagebrush, whereas (panel c) illustrates an area where restoration is prioritized because there are large patches of degraded sagebrush where economy of scale could yield large gains for focal species habitat relative to restoration investment. (Panels b and d) indicate whether optimization solutions were sensitive to uncertainty in the spatial input data in the corresponding areas, potentially influencing the restoration planning process with regard to decision‐makers' risk tolerance.

Restoring 20% of degraded sagebrush habitat for sagebrush‐obligate and ‐associated species across Wyoming required almost 200,000 ha and could potentially increase the amount of intact sagebrush hosting focal species' populations by 5% (sage thrasher) to 8% (green‐tailed towhee; Table 2). There were considerable differences in the spatial configuration of potential restoration sites between areas with relatively intact landscapes, where selected sites were more dispersed (Figure 5a), and more degraded areas, where selected sites were clustered (Figure 5c). Values of potential species' feature layers selected by problem 2a (high resilience) were generally lower than values of feature layers selected by problem 1a (no cost) but similar to values across intact sagebrush (Figure S4 in Data S2). Predicted future sagebrush cover was similar between sites selected by problem 2a (high resilience) and problem 1a (no cost), while spring soil moisture availability was slightly greater and lek connectivity was slightly lower for sites selected by problem 2a (Figure S5 in Data S2).

In some regions, problem 2b (uncertainty) selected sites repeatedly across problems, despite increasing uncertainty penalties (Figure 5b), while other areas were substantially more sensitive to uncertainty in the spatial data inputs (Figure 5d). To illustrate these differences, we compared sites selected in ≥ 7 iterations (low sensitivity), 4 to 6 iterations (medium sensitivity), and ≤ 3 iterations (high sensitivity). Compared to sites with low sensitivity to uncertainty, sites that were highly sensitive to uncertainty had lower median densities of Brewer's sparrow, green‐tailed towhee, and sage thrasher, as well as lower median probability of pygmy rabbit presence, but similar median probability of greater sage‐grouse lek persistence and greater median densities of sagebrush sparrow (Figure S4 in Data S2). Lek connectivity decreased with increasing sensitivity to uncertainty, and sites with low sensitivity had greater spring soil moisture availability and future sagebrush cover than sites with medium to high sensitivity (Figure S5 in Data S2).

### 4.3 Problem Set #3: Enhancing Existing Conservation Strategies

**FIGURE 6:**
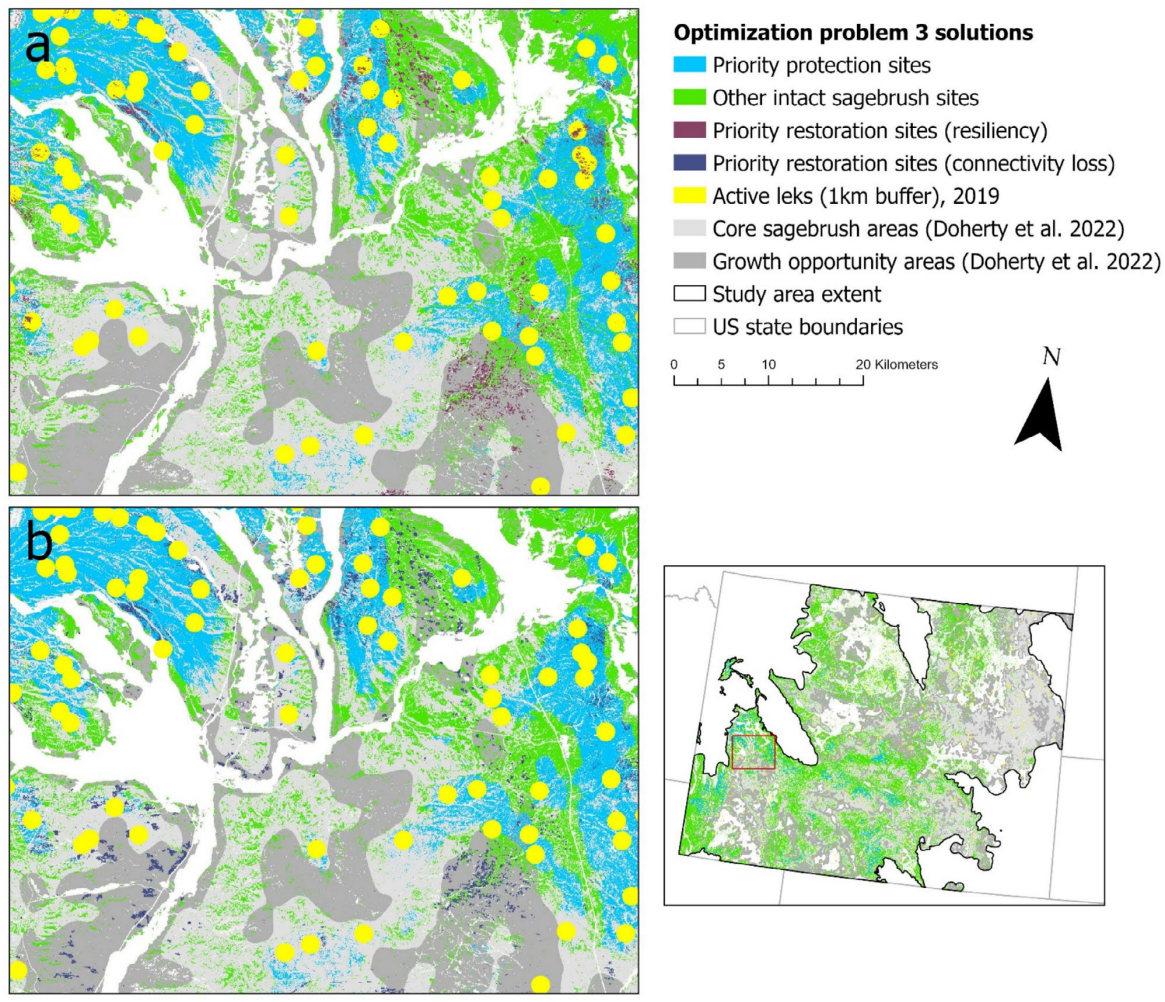
Solutions for optimization problem set #3, identifying priority protection and restoration sites to complement the Sagebrush Conservation Design (SCD; Doherty et al. 2022) by informing finer‐scale sagebrush management in Wyoming. These multi‐species optimization solutions prioritized sites for protection of intact sagebrush sites by maximizing greater sage‐grouse ( *Centrocercus urophasianus* ) lek connectivity within SCD‐identified core sagebrush and growth opportunity areas (panels a and b). Restoration was prioritized by maximizing resilience (i.e., likelihood of restoration success; panel a) or lek connectivity lost over the past 35 years (panel b). The figure panels provide context for where priority sites fall within core sagebrush and growth opportunity areas, as well as the 1‐km protection buffers surrounding current, occupied greater sage‐grouse leks (as of 2019). By identifying areas of highest priority, based on user‐specified constraints and species targets, these solutions can further support existing conservation strategies by providing data‐driven, supplemental resources to focus sagebrush conservation and restoration action.

Limiting protection of sagebrush‐obligate and ‐associated species to intact sagebrush within CSAs and GOAs identified by the SCD generated finer‐scale priority maps for conservation delivery within these larger areas (Figure 6). Sites selected for the protection of intact sagebrush (problem 3a, SCD protection) were strongly clustered within CSAs (89.42% overlap), while sites selected for the restoration of degraded sagebrush overlapped CSAs to lesser extents (64.65% for 3b (SCD resilience) and 54.59% for 3c (SCD connectivity)).

Intact sagebrush sites selected by problem 3a (SCD protection) supported similar densities and probabilities of focal species to those within sites selected for protection without constraint by the SCD (problem 1b, connectivity). However, sites selected by problem 3a (SCD protection) supported lower densities of Brewer's sparrow and green‐tailed towhee and greater densities of sagebrush sparrow (Figure S6 in Data S2). Except for sagebrush sparrow, values of species' feature layers were also consistently higher across sites selected in problems 1b (connectivity) and 3a (SCD protection) than across CSAs, Wyoming core areas, and intact sagebrush sites across Wyoming (Figure S6 in Data S2). Spring soil moisture availability and future sagebrush cover were lower for sites selected by problem 3a (SCD protection) than for sites selected by problem 1b (connectivity) and higher for lek connectivity. Lek connectivity was also higher in sites selected by 3a (SCD protection) than on average across CSAs (Figure S7 in Data S2).

Restoration problems integrated into the SCD (3b (resilience) and 3c (connectivity)) selected sites with similar or greater values of species' feature layers than sites selected for restoration without constraint by the SCD (problem 2a (high resilience)), except for problem 3c (SCD connectivity), selecting sites with lower densities of Brewer's sparrows (Figure S6 in Data S2). Sites selected by problem 3b (SCD resilience) also showed similar or greater values for cost layers to sites selected by problem 2a (high resilience), while problem 3c (SCD connectivity) selected sites with lower spring soil moisture availability and future sagebrush cover. Problem 3b (SCD resilience) selected sites with greater spring soil moisture availability and future sagebrush cover than CSAs and GOAs, while problem 3c (SCD connectivity) selected sites with greater lek connectivity than CSAs and GOAs (Figure S7 in Data S2).

## 5 Discussion

The maps generated by our optimization problems represent mathematically optimal solutions that can serve as decision‐support resources for land managers and other practitioners working within Wyoming's sagebrush steppe. More broadly, we demonstrated an SCP framework that integrated protection and restoration planning, explored trade‐offs between multi‐ and single‐species optimizations and between prioritizing different ecological constraints (e.g., connectivity, restoration feasibility), explicitly assessed uncertainty in data inputs, and advanced existing, broad‐scale conservation plans. When combined with other available data and local expertise, these maps can assist decision‐makers in allocating limited conservation resources to maximize the effectiveness and efficiency of their investments. While we demonstrated the application of our tool by addressing specific conservation challenges across sagebrush ecosystems in Wyoming, the particular inputs, their configuration, and the tool development process itself are flexible and can be adapted to incorporate novel input data (e.g., Holdrege et al. 2024; Theobald et al. 2024) or prioritize meeting other management objectives. Here, we discuss key considerations and trade‐offs regarding SCP tools in hopes of providing guidance that can facilitate the development of actionable tools that meet stakeholder needs across a broad array of ecological systems, geographic regions, species, and management questions.

### 5.1 Balancing the Needs of Multiple Species

Greater sage‐grouse have been promoted as an umbrella species for the conservation of sagebrush‐dependent and ‐associated species (Rowland et al. 2006), and there is evidence an umbrella species approach could provide benefits for some sagebrush‐obligates (Hanser et al. 2011; Donnelly et al. 2017; Smith et al. 2019). Optimizing protection for greater sage‐grouse alone was more successful at protecting non‐target species than all other species investigated (although Brewer's sparrow and pygmy rabbit, and to a lesser extent sage thrasher, provided considerable protection to some other species; Table 2). However, this was largely due to the greater area required to achieve relative (or proportional) protection targets for greater sage‐grouse. While we initially interpreted the larger area selected by the single‐species greater sage‐grouse problem as evidence of sage‐grouse's requirement for large expanses of intact habitat (Aldridge et al. 2008; Connelly et al. 2011), it was actually an artifact of differences in the data types we used to prioritize focal species protection. Sagebrush songbirds were prioritized using density estimates, while greater sage‐grouse were prioritized using lek persistence probability because these represented the best species‐specific data layers available for the application. Density estimates exhibit a naturally right‐skewed Poisson distribution, whereas lek persistence probability follows a binomial distribution that is left‐skewed, partly due to the high likelihood of lek persistence across Wyoming (Wann et al. 2023). As a result, the highest values of songbird densities were much greater than the mean, allowing *prioritizers* to meet the target of protecting 20% of feature values by selecting fewer pixels than needed to satisfy that target for greater sage‐grouse, where the highest available values were much closer to the mean. This effect highlights the importance of investigating intuitive explanations of model results, as well as careful consideration of input data used in optimization problems. For example, if stakeholders wanted the SCP tool to treat greater sage‐grouse and sagebrush songbirds equally, this problem could be accounted for by reducing relative targets in proportion to the distribution effects (e.g., approximately a 50% reduction in this case; J. O. Hanson, Carleton University, written comm., May 1, 2024). This effect also likely exaggerated the efficacy of using greater sage‐grouse as an umbrella species in our results by inflating the area needed to meet conservation targets. However, this effect is partly driven by the high probability of lek persistence in Wyoming sagebrush ecosystems, and the success of Wyoming core areas in preserving habitat for other sagebrush‐dependent species highlights the value of using greater sage‐grouse as an umbrella for many species, despite its limitations (Table 2; Aldridge et al. 2024).

Despite the increased area selected as a result of using lek persistence probability as an indicator, the single‐species greater sage‐grouse problem failed to provide an umbrella for one of the focal species (sagebrush sparrow), similar to other studies showing that using greater sage‐grouse as an umbrella species can lead to insufficient protection for some sagebrush‐associated species of conservation concern (e.g., Rowland et al. 2006; Copeland et al. 2014; Carlisle et al. 2018; Aldridge et al. 2024). We found that explicit consideration of multiple species effectively met protection targets for each species (Table 2) and required a similar total area compared to the greater sage‐grouse solution (549,435 vs. 548,359 ha, respectively). While these results are also influenced by the left‐skewed distribution of lek persistence probability, the minor increase (0.2%) in area needed to collectively meet all species' targets compared to meeting those targets individually suggests the cost to optimize management for all focal species instead of a partially effective umbrella species could be negligible. However, meeting protection targets for all focal species required trade‐offs when compared to single‐species problems. For example, most focal species in our study are positively associated with sagebrush cover (Smith et al. 2019; Van Lanen et al. 2023b; Wann et al. 2023), but sagebrush sparrows prefer low‐elevation open shrublands, avoiding areas of dense sagebrush (Timmer et al. 2019; Van Lanen et al. 2023b). Consequently, meeting protection targets for sagebrush sparrow necessitated selection of sites with lower feature layer values for some species (Figure S1 in Data S2), particularly green‐tailed towhees, which are associated with dense sagebrush at higher elevations (Van Lanen et al. 2023b). Similarly, Wyoming core areas protected a lower proportion of sagebrush sparrow populations than other focal species (Table 2). The mismatch between these species' habitat associations highlights the need for SCP developers to think carefully about the habitat requirements of their focal species relative to their management priority (Carlisle et al. 2018; Duchardt et al. 2021). For example, if the sagebrush sparrow or green‐tailed towhee were considered a lower management priority, their protection targets could be lowered accordingly (e.g., 15%). These results have important implications for ecosystem management, especially when considering ecotones (e.g., areas of overlap between sagebrush and pinyon‐juniper woodlands) where managing for umbrella species could be detrimental to non‐target species of concern (Duchardt et al. 2023; Van Lanen, Duchardt, et al. 2024). For example, pinyon jays ( *Gymnorhinus cyanocephalus* ) are increasingly recognized as a species of high conservation priority (Boone et al. 2018) that share space with many species in our study but have potentially competing habitat requirements that tools like PReSET are well‐suited to addressing (Van Lanen, Shyvers, et al. 2023).

### 5.2 Incorporating Ecological Constraints and Uncertainty Into Optimization Problems

Overall, results of our optimization problems illustrated the capacity for SCP tools to effectively inform species conservation planning in ways that integrate specific management goals such as preserving connectivity or managing for resilience given changing climatic conditions. However, the sensitivity of our results to cost layer inputs (e.g., connectivity, future sagebrush cover projections; Figure 3) emphasizes that careful consideration is needed when choosing focal ecological constraints. For example, sites selected by problem 1d (low resilience) had considerably lower spring soil moisture availability than those selected by other protection problems (i.e., problem set #1). Similarly, problem 1c (future sagebrush) selected sites with the greatest estimates of future sagebrush cover, and sites selected by problem 1b (connectivity) had slightly greater lek connectivity than other iterations of problem 1 (Figure S3 in Data S2). Interestingly, we found that problem 1d (low resilience) also selected sites with greater predicted pygmy rabbit presence, sage thrasher density, and sagebrush sparrow density than other iterations of problem 1. At the same time, these sites had lower greater sage‐grouse lek persistence probability, Brewer's sparrow density, and green‐tailed towhee density (Figure S2 in Data S2). Ecological differences in selected sites may thus translate into differences in habitat quality, with groupings reflecting preferences for more arid habitats located at lower elevations for the former species (Smith et al. 2019; Van Lanen et al. 2023b) and more mesic habitats located at higher elevations for the latter (Van Lanen et al. 2023b, Wann et al. 2023). The strong influence of cost layers on optimization outcomes makes it critical that SCP developers work closely with end‐users to verify cost layers represent important limiting factors for management (e.g., restoration feasibility, dollar cost). If the influence of cost layer values on optimization solutions generates unrealistic results for guiding management, this problem can be ameliorated by modifying planning units or cost layers to better reflect their influence on management objectives. For example, excluding sites with less than 10% future sagebrush cover from the planning unit layer for problem 1d (low resilience) resulted in cost and feature layer values more similar to the other iterations of problem 1. SCD developers can also include additional costs as penalty layers that use a selected scaling factor (Hanson et al. 2021) to multiply the cost of a given pixel (e.g., spring soil moisture as an indicator of restoration success) by (an)other constraint(s) of interest (e.g., dollar cost of restoration treatments). Such preprocessing of input layers or reconfiguring of prioritization problems could thus help SCP developers design problems that better balance priorities to meet multiple management objectives.

Boundary penalties are another way to apply constraints to optimization problems (Ardron et al. 2010). Our use of boundary penalties was intended to cluster selected pixels so they could serve as actionable project areas and to incorporate information on spatial configuration in the surrounding landscape (e.g., prioritizing restoration sites adjacent to intact sagebrush). Accordingly, in problem 2a (high resilience), selected restoration sites were often dispersed across intact sagebrush landscapes where small‐scale restoration could have a high probability of success or aggregated over more degraded regions where larger‐scale restoration efforts could realize higher returns on investment by concentrating efforts and increasing efficiency. However, the effect of boundary penalties is proportional to the combination of input data used in any given problem, making calibration a potentially challenging process (Ardron et al. 2010; Hanson et al. 2021). Furthermore, boundary penalties can create a trade‐off where the effort to reduce overall boundary length, and thereby clustering selected sites, can lead to the selection of sites with lower feature layer values (Hanson et al. 2021, 2022). For example, sites selected by problem 2b (uncertainty) supported higher values for most focal species' feature layers than sites selected under problem 2a (high resilience), possibly due to the absence of boundary penalties in problem 2b (uncertainty). Boundary penalties are often used as a surrogate for habitat connectivity (Hanson et al. 2022), yet we found that sites selected using a boundary penalty (e.g., problem 2a: high resilience) did not exhibit greater lek connectivity than sites selected without a boundary penalty (e.g., problem 2b: uncertainty), highlighting previous research demonstrating that boundary penalties are not ideal for prioritizing connectivity (Hanson et al. 2022). Indeed, we found the only problems that increased lek connectivity were those specifically using modeled estimates of connectivity as a cost layer (i.e., problems 1b (connectivity) and 3a (SCD protection)). This highlights the need for SCP developers to consider whether their objective is to aggregate selected sites for management or other logistical purposes, or to select sites with high functional connectivity of habitats, rather than just adjacency or clustering of selected sites.

Finally, incorporating uncertainty into SCP optimizations remains challenging for conservation planning. Several studies attempted to address uncertainty in SCP applications, for example, by applying greater weight to areas more likely to remain suitable for species in the future (Moilanen et al. 2024), penalizing predicted occurrence probabilities based on associated measures of uncertainty (Kujala et al. 2013), and using principles of Modern Portfolio Theory to quantify risk and employ diversification as a means to manage it (e.g., by quantifying risk as the variance and correlation among assets to facilitate risk management in climate‐driven reserve design; Eaton et al. 2019). We created a potential workflow for quantifying uncertainty in a visually and spatially explicit manner by generating a map illustrating which prioritized sites are more (or less) sensitive to data uncertainty (Figure 5). Land managers frequently cite a lack of trust in data products as a barrier to using them (Diez and McIntosh 2011; Van Lanen, Shyvers, et al. 2024), but transparency about the uncertainty of data informing these products can build trust (McInerny et al. 2014; Nel et al. 2016). Low sensitivity in this map (Figure 5) indicates sites where either the uncertainty of input layers was low, or moderate uncertainty was offset by very high feature layer values (or very low‐cost layer values). Managers could use this map directly or overlay it with outputs of other optimization problems to assess which priority sites match their personal or organizational level of risk tolerance.

### 5.3 Integrating Existing Conservation Plans Into SCP Efforts

Decision‐support frameworks and tools may be of limited value if they do not incorporate current policies or ongoing conservation efforts. For this reason, we tested the efficacy of integrating an existing regional conservation strategy, the SCD (Doherty et al. 2022), into SCP applications using our expanded tool. The SCD was developed using key vegetation and human development metrics to categorize the landscape into CSAs, GOAs, and other rangeland areas. These designations were aimed at coordinating management across the biome into a strategic and cohesive effort to “defend and grow the core” (Western Governors' Association 2020; Doherty et al. 2022). However, CSAs and GOAs are often large, heterogeneous patches of habitat, meaning that sites within any given CSA or GOA may require different management actions or provide habitat for different species. For example, while sites selected for restoration were more likely to be located within GOAs than sites selected for protection, most restoration sites occurred within CSAs, illustrating that degraded sagebrush habitats may still occur in core sagebrush habitats and could represent especially high‐priority restoration opportunities due to the presence of more favorable surrounding conditions (Boyd et al. 2024). Likewise, many sites were selected for protection in GOAs, suggesting there may be patches of core‐equivalent habitats within GOAs that could benefit from habitat protection to ‘defend the core’ or serve as nuclei for adjacent restoration work. Consistent with recent findings (Kumar et al. 2024; Prochazka et al. 2024), our tool preferentially selected sites for both protection and restoration in CSAs compared to GOAs, highlighting the value of CSAs for providing important songbird and sage‐grouse habitat. Yet sites selected within CSAs and GOAs in problem 3 also supported densities and probabilities of focal species that were greater than average values across all CSAs and GOAs, demonstrating the capability of our tool to guide finer resolution targeting of habitat management. Similarly, sites selected by our tool also had higher soil moisture availability values than average conditions across CSAs and GOAs, suggesting greater resilience to disturbance and climate change. This was unsurprising because the SCD did not specifically include indicators of resilience in its classifications (Doherty et al. 2022). However, this highlights opportunities for SCP tools like PReSET to target sites within existing regional‐scale or statewide plans to meet emerging management objectives based on factors such as multi‐species metrics, habitat connectivity, restoration feasibility, or areas of possible climate refugia. While we only investigated applications at this larger, regional scale to help support cross‐jurisdictional management efforts, our tool could also be applied at smaller extents to directly address local management efforts (Van Lanen, Shyvers, et al. 2024) or across multiple scales to investigate trade‐offs and consistencies among identified priority management sites.

### 5.4 Limitations and Considerations

We identified several important considerations for interpreting and applying optimization solutions generated by our tool's current framework and spatial data inputs. First, the results of our optimization problems are intended to guide conservation planning in tandem with local expertise and data. Individual 30‐m pixels from our maps may not be independently actionable, and indeed, the input layers we used to optimize the selection of pixels were intended for broader‐scale applications (e.g., Rigge et al. 2022; Wann et al. 2023). End‐users could thus base implementation decisions on broader spatial patterns (i.e., areas with clusters of selected sites) augmented with local knowledge or ground truthing, rather than on a pixel‐by‐pixel basis. Developers of SCP could also aggregate selected sites in more management‐relevant configurations (e.g., by using boundary penalties), as we did in problem 2a (high resilience). Additionally, selected sites could be used to evaluate multiple potential project areas. For example, end‐users could compare the proportion of selected pixels in candidate project areas to decide which areas are more suitable for meeting management objectives.

Second, planning units have an outsized influence on prioritization results because they represent a binary decision on whether sites will be considered by the optimization process or not. For our planning units, we used sagebrush cover estimates and classified them as intact or degraded sagebrush habitats. Thus, our solutions could exclude important conservation areas that did not meet our definitions of intact (≥ 15% remaining sagebrush cover) or degraded (significant decrease in sagebrush over time) sagebrush. For example, sagebrush habitats in northeastern Wyoming are unlikely to meet this threshold (Gordon 2019), and as a result, may be underrepresented in sites selected for protection of ‘intact’ habitat by our tool.

Third, we evaluated spatially explicit conservation benefits of restoration by assuming key wildlife metrics would revert to mean values for intact sagebrush within ecologically meaningful regions (i.e., sage‐grouse population clusters). Although this approach represents a reasonable approximation of expected conservation returns, direct simulations of sagebrush restoration at each site could provide more precise inference regarding expected wildlife responses (refer to Orning et al. 2023).

## 6 Conclusions

SCP is a popular and useful component of systematic conservation planning needed to inform biodiversity protection in a rapidly changing global environment (Cobb et al. 2024). Here, we extended the functionality of a preliminary SCP tool by linking prioritization of protection and restoration actions to support holistic, broad‐scale decision‐making within vast and heterogeneous biomes that are increasingly threatened. We leveraged spatial data representing connectivity, future climate projections, and ecosystem resilience to gauge multi‐taxa conservation outcomes and potential impacts of data uncertainty on management returns, demonstrating the flexibility of SCP to meet a diverse set of management goals and explore associated trade‐offs and synergies. Importantly, we found that SCP could:

1. Improve upon imperfect umbrella and other single‐species approaches to conservation at a minimal cost in conservation investments.
2. Meet ecological management objectives (e.g., restoration feasibility) while also meeting focal species' conservation objectives and explicitly illustrating uncertainty regarding management objectives.
3. Drill into coarse‐scale conservation plans to target optimal sites for delivery of specific management actions.

However, a persistent theme across optimization problems and input data types was that input data strongly influenced the selection of priority sites, highlighting the need to carefully consider which input data are included. Alignment of input data and their configuration in optimization problems (e.g., as a cost layer vs. feature layer) with management objectives is particularly important (Evans et al. 2015) and can be improved through the coproduction of SCP tools (Beier et al. 2017). Furthermore, there is a need for continued advances in spatial ecology and the development of species and environmental data layers that can be easily incorporated into large‐scale conservation frameworks to help guide broad‐reaching conservation efforts. As these data emerge, PReSET could be expanded to incorporate economic costs of management actions, prioritize alternative restoration actions (e.g., invasive annual grass control, prescribed fire, mechanical tree removal, or sagebrush planting), consider socio‐economic outcomes, mitigate risks of anthropogenic development or disturbance (e.g., wildfire, spread of invasive species, or infrastructure siting), integrate additional taxa, and more.

As advances in software, input data, and workflows increase the breadth of considerations that can be incorporated into SCP tools, so will the burden on conservation planners and decision‐makers in selecting inputs, designing problem sets, and interpreting solutions. This is especially pertinent for large‐scale conservation planning efforts, which inherently involve a greater number and diversity of potential stakeholders, each with unique information needs and management objectives. Thus, large‐scale conservation planning applications will require more communication and coordination among stakeholders for holistic and effective conservation planning to occur (Van Lanen, Shyvers, et al. 2024). There is an urgent need for direct guidance on engaging potential end‐users to facilitate coproduction of large‐scale SCP tools and frameworks (Van Lanen, Shyvers, et al. 2024). Such guidance could demonstrate how SCP developers can proactively coordinate with stakeholders from diverse entities and organizational levels (from program leadership governing funding allocation to local biologists directing on‐the‐ground management), leading to more actionable science and effective outcomes.

## Author Contributions

**Jessica E. Shyvers:** conceptualization (lead), data curation (lead), formal analysis (equal), methodology (lead), project administration (lead), resources (lead), writing – original draft (lead), writing – review and editing (equal). **Bryan C. Tarbox:** conceptualization (supporting), data curation (supporting), formal analysis (equal), methodology (supporting), project administration (supporting), resources (supporting), writing – original draft (equal), writing – review and editing (lead). **Adrian P. Monroe:** conceptualization (supporting), data curation (supporting), methodology (supporting), resources (supporting), writing – review and editing (supporting). **Nicholas J. Van Lanen:** conceptualization (supporting), data curation (supporting), methodology (supporting), writing – review and editing (supporting). **Benjamin S. Robb:** conceptualization (supporting), methodology (supporting), resources (supporting), writing – original draft (supporting), writing – review and editing (supporting). **Erin K. Buchholtz:** data curation (supporting), methodology (supporting), resources (supporting), writing – review and editing (supporting). **Courtney J. Duchardt:** conceptualization (supporting), methodology (supporting), writing – review and editing (supporting). **David R. Edmunds:** conceptualization (supporting), data curation (supporting), methodology (supporting), resources (supporting), writing – review and editing (supporting). **Michael S. O'Donnell:** conceptualization (supporting), data curation (supporting), methodology (supporting), resources (supporting), writing – review and editing (supporting). **Nathan D. Van Schmidt:** data curation (supporting). **Julie A. Heinrichs:** conceptualization (supporting), writing – review and editing (supporting). **Cameron L. Aldridge:** conceptualization (supporting), data curation (supporting), funding acquisition (lead), methodology (supporting), project administration (supporting), resources (supporting), supervision (lead), writing – review and editing (supporting).

## Conflicts of Interest

The authors declare no conflicts of interest.

## Supporting information


**Data S1:** ece372214‐sup‐0001‐DataS1.docx.

**Data S2:** ece372214‐sup‐0002‐DataS2.docx.

## Data Availability

Datasets generated as part of this study are available via a USGS ScienceBase data release (Shyvers et al. 2025) and can be accessed at https://doi.org/10.5066/P14TYNTY.
