# Supplementary material for: Different Data for Different Goals: Exploring Trade‐Offs and Synergies in the Use of Spatial Data Inputs to Optimize Conservation Action in Sagebrush Ecosystems
Source: Ecol Evol. 2025 Sep 30;15(10):e72214. doi: 10.1002/ece3.72214 (PMC12483841; doi:10.1002/ece3.72214)
Supplement: Supplementary file 1 — Data S1: ece372214‐sup‐0001‐DataS1.docx. [file ECE3-15-e72214-s002.docx]

Supplemental A: Methods and products supporting the manuscript ***“Different data for different goals: exploring trade-offs and synergies in the use of spatial data inputs to optimize conservation action in sagebrush ecosystems”***

Jessica E. Shyvers^1,2*^ (ORCID: 0000-0002-4307-0004), Bryan C. Tarbox^1^ (0000-0001-5040-3949), Adrian P. Monroe^1^ (0000-0003-0934-8225), Nicholas J. Van Lanen^1^ (0000-0003-0871-0261), Benjamin S. Robb^1,3^ (0000-0003-1419-3918), Erin K. Buchholtz^1,4^ (0000-0002-1985-9531), Courtney J. Duchardt^5^ (0000-0003-4563-0199), David R. Edmunds^1^ (0000-0002-5212-8271), Michael S. O’Donnell^1^ (0000-0002-3488-003X), Nathan D. Van Schmidt^1,6^ (0000-0002-5973-7934), Julie A. Heinrichs^7^ (0000-0001-7733-5034), Cameron L. Aldridge^1^ (0000-0003-3926-6941)

^1^U.S. Geological Survey, Fort Collins Science Center, 2150 Centre Ave, Bldg. C, Fort Collins, CO 80526

^2^The Nature Conservancy, Protect Oceans, Land and Water. Fort Collins, CO (current affiliation)

^3^Department of Zoology and Physiology, University of Wyoming, Laramie, WY (current affiliation)

^4^U.S. Geological Survey, South Carolina Cooperative Fish and Wildlife Research Unit, Clemson University, Clemson, SC (current affiliation)

^5^School of Natural Resources and the Environment, University of Arizona, Tucson, AZ 85721 (current affiliation)

^6^San Francisco Bay Bird Observatory, 524 Valley Way, Milpitas, CA 95035 (current affiliation)

^7^Natural Resource Ecology Laboratory, Colorado State University, Fort Collins, CO 80523 in cooperation with the U.S. Geological Survey, Fort Collins Science Center, 2150 Centre Ave, Bldg. C, Fort Collins, CO 80526

*Corresponding Author: jess.shyvers@tnc.org, 970-556-8119

**Development of planning unit layers (degraded sagebrush, intact sagebrush)**

To develop planning units for protection and restoration problems, we first created a data layer mapping degraded sagebrush. We calculated the mean value of sagebrush cover across 2019-2020 (Rigge et al. 2022), then subtracted it from the mean value of sagebrush cover across 1985-1986. This resulted in a layer depicting change in sagebrush cover from 1985-1986 to 2019-2020, with positive values representing amount of sagebrush cover lost. We then subtracted the mean 2019-2020 sagebrush cover estimates from the ecological potential estimates derived by Rigge et al. (2021) to identify sites where sagebrush cover may have been lost or degraded prior to 1985. We then reclassified each of these layers where:

1: < (mean change + 1SD)

2: => (mean change + 1SD) and < (mean change + 2SD)

3: => (mean change + 2SD)

We then added these two raster layers and reclassified them again, where:

2-3 = 0

4-6 = 1

Thus, our degraded sagebrush layer (Figure SA1) includes all sites where sagebrush loss was at least two standard deviations above mean change from historic or potential estimates, or where both divergence from historic and potential estimates were between one and two standard deviations above the mean. We then subtracted our degraded sagebrush layer from current estimates (2019-2020) of sagebrush cover that met or exceeded 15% cover to generate a layer of intact sagebrush (Figure SA2).


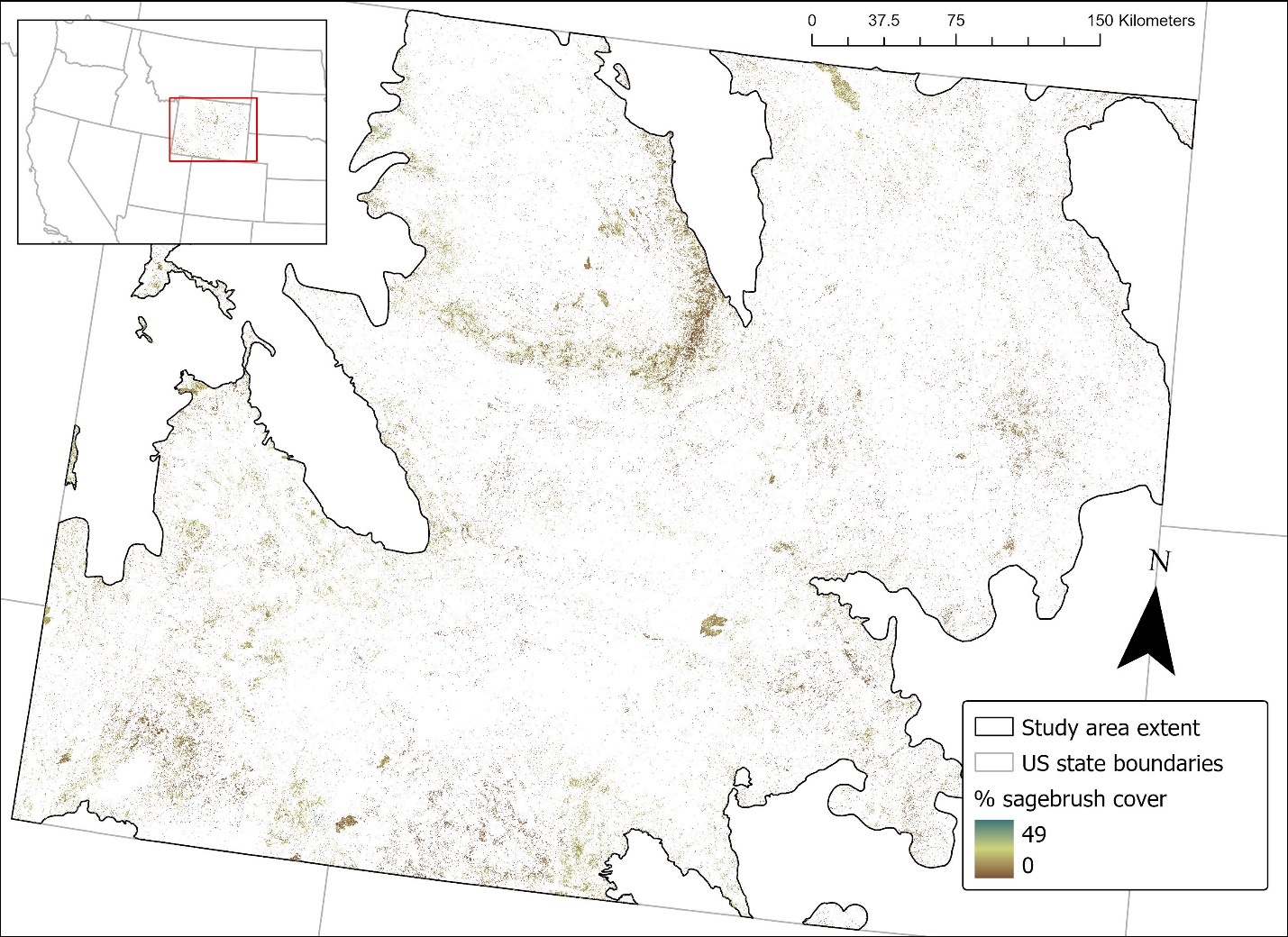


**Figure SA1:** Map of degraded sagebrush sites in Wyoming, USA. 30-meter resolution pixel values represent 2020 percent sagebrush fractional cover estimates from Rigge et al. (2022). Study area extent approximates the sagebrush biome within the state.


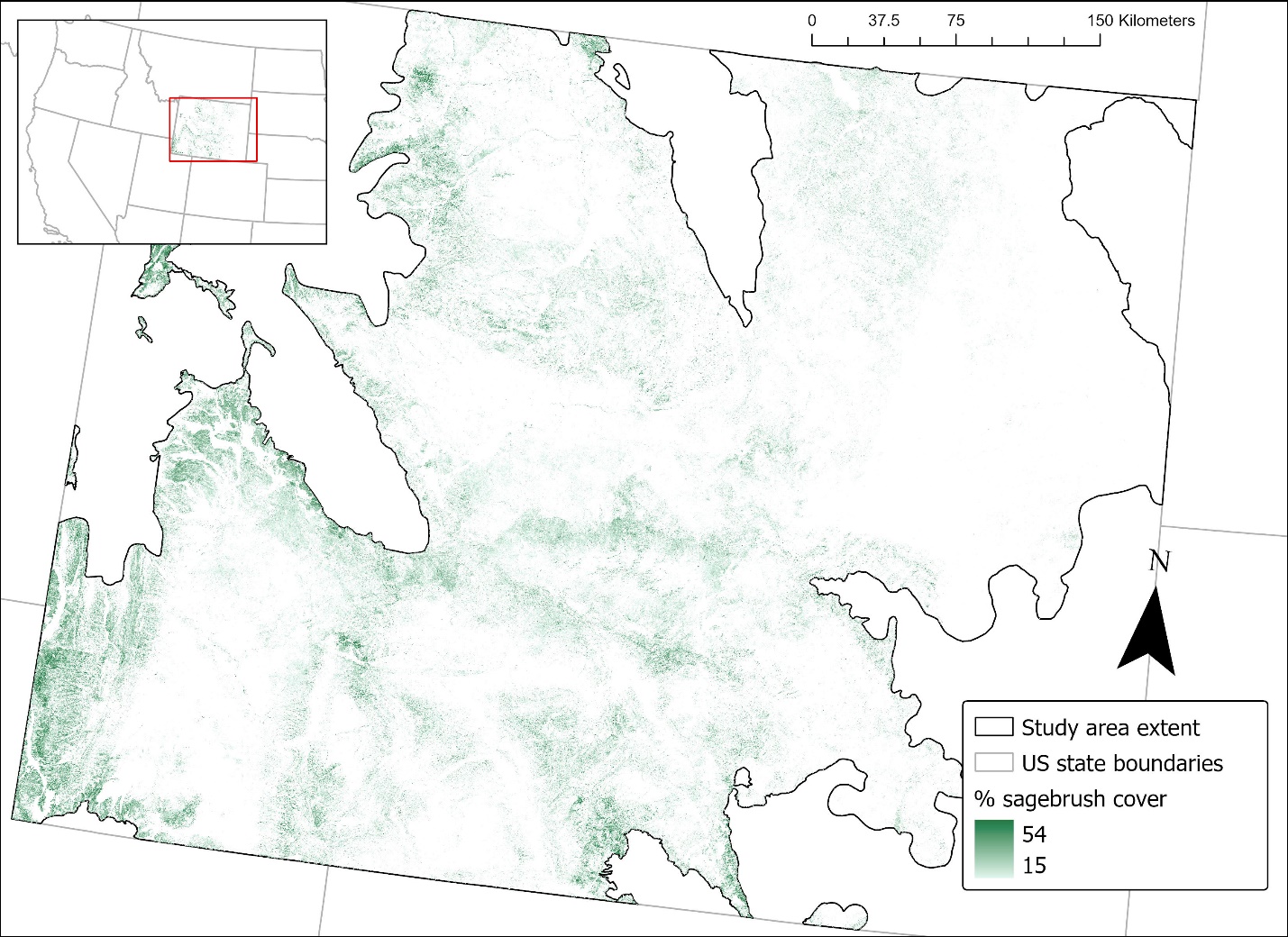


**Figure SA2:** Map of intact sagebrush sites in Wyoming, USA. 30-meter resolution pixel values represent 2020 percent sagebrush fractional cover estimates from Rigge et al. (2022). Study area extent approximates the sagebrush biome within the state.

**Development of connectivity cost layers (sagebrush connectivity, lek connectivity (2020), and lek connectivity loss (1985 to 2020))**

*Omnidirectional circuit-based approach*

Circuit-based connectivity modeling has a wide range of connectivity applications (Dickson et al. 2019). Circuit theory uses current density for electrical flow as a proxy for potential connections among sources and grounds and the flow is determined by resistance (McRae and Beier 2007; McRae et al. 2008). The flow of electricity is analogous to a random-walk model and considered a valuable model for wildlife movement and other landscape connections where random-walk paths are appropriate (Dickson et al. 2019). Connectivity can be used to understand the potential physical linkages among patches of habitat from a structural perspective (i.e., does vegetation structure generally allow or impede wildlife movement), or with added species-specific information can be used to inform a more functional understanding of potential connections for particular wildlife species.

We used an omnidirectional circuit-based approach (McRae et al. 2016; Landau et al. 2021) to model both structural connectivity (i.e., sagebrush connectivity) and functional connectivity (i.e., lek connectivity) for the state of Wyoming. The primary components for the omnidirectional approach included the conductance surface (the inverse of resistance), an input representing current sources and targets, and parameters for the size of moving window within which current density would be calculated in connecting those sources and targets (Landau et al. 2021). Refer to <https://docs.circuitscape.org/Omniscape.jl/stable/> for more detailed information on the Omniscape algorithm and how it works.

We modeled three connectivity surfaces: sagebrush connectivity, lek connectivity (2020), and lek connectivity (1985). We used lek connectivity surfaces to calculate change in connectivity among leks over time (i.e., lek connectivity loss (1985 to 2020)). We present maps of sagebrush connectivity (Figure SA4) and lek connectivity loss (1985 to 2020; Figure SA5) below.

*Input data and parameters for modeling connectivity*

Conductance surface: We used the fractional component of sagebrush at 30-m resolution (RCMAP v3; Rigge et al. 2022) for the conductance surface inputs for each connectivity model. For sagebrush connectivity and lek connectivity (2020) we used sagebrush cover estimates from 2020, and for lek connectivity (1985) we used sagebrush cover estimates from 1985. This resulted in greater resistance in areas with no or low sagebrush cover, and less resistance in areas with high sagebrush cover. We did not include any impermeable barriers or make assumptions for conductance of other landscape features beyond sagebrush cover. We included a 30 km buffer around the study area to remove potential edge effects, which was then masked out of the final output.

Sources: The sources input determines the location and strength of the current flow. Each moving window iteration is centered on a single source pixel, and the current density is calculated among that center (target) pixel and the surrounding source pixels within the window (see Figure SA3). The amount of current is based on the strength of the target and source pixels. The source layer itself can be based on a subset version of the conductance surface, whereby any pixels over a certain threshold are considered sources (i.e., as in our sagebrush connectivity model), or alternatively by supplying a separate source raster (i.e., as in our lek connectivity models). For sagebrush connectivity, source pixels were selected as those with moderately abundant sagebrush cover (>15% fractional component) based on the 2020 sagebrush fractional component raster for the source layer (RCMAP v3; Rigge et al. 2022). For lek connectivity, we limited source and target pixels to the locations of known, active leks during 2010-2019 (O’Donnell et al. 2021) and weighted all leks equally (AMP = 1).

Moving window: For all models, we calculated cumulative current density outputs using a 30-km moving window. This window represented a large but possible distance for greater sage-grouse movement. See Figure SA3 for illustration of how the moving window bounds the current density calculation.

| 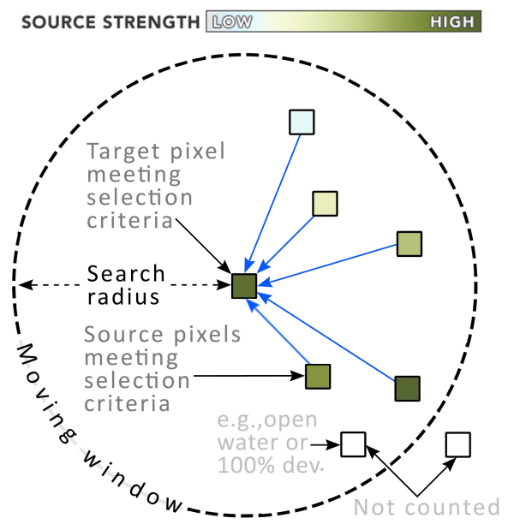 | **Figure SA3.** An illustration of a moving window iteration in the Omniscape algorithm.  *Source:* [*https://docs.circuitscape.org/Omniscape.jl/stable/algorithm/#The-Omniscape-Algorithm*](https://docs.circuitscape.org/Omniscape.jl/stable/algorithm/#The-Omniscape-Algorithm) |
| --- | --- |

*Computation*

For computational efficiency we divided the study area into tiles. We included 30-km buffer overlap for the tiles that would eliminate potential edge effects from the moving window calculations; the buffered areas were then removed and the tiles mosaicked together. We used the Omniscape software (v 0.5.7, Landau et al. 2021) in Julia (v 1.7.2; Bezanson et al. 2017; Hall et al. 2021). We also used a block size of 105 in the Omniscape initialization files to reduce computational needs. Omniscape was run on the USGS Denali Supercomputer (Falgout et al. 2022). All raster processing was conducted using R programming language (R Core Team 2021).

*Calculating lek connectivity loss*

We calculated the cumulative current density for contemporary lek connectivity (2020), and historical lek connectivity (1985). To represent the loss in potential connectivity over time, we subtracted the 2020 current density raster from the 1985 raster. Because increases in connectivity measures may be the result of redirected current and not actual increases in sagebrush, we set all connectivity change values > 0 equal to zero.


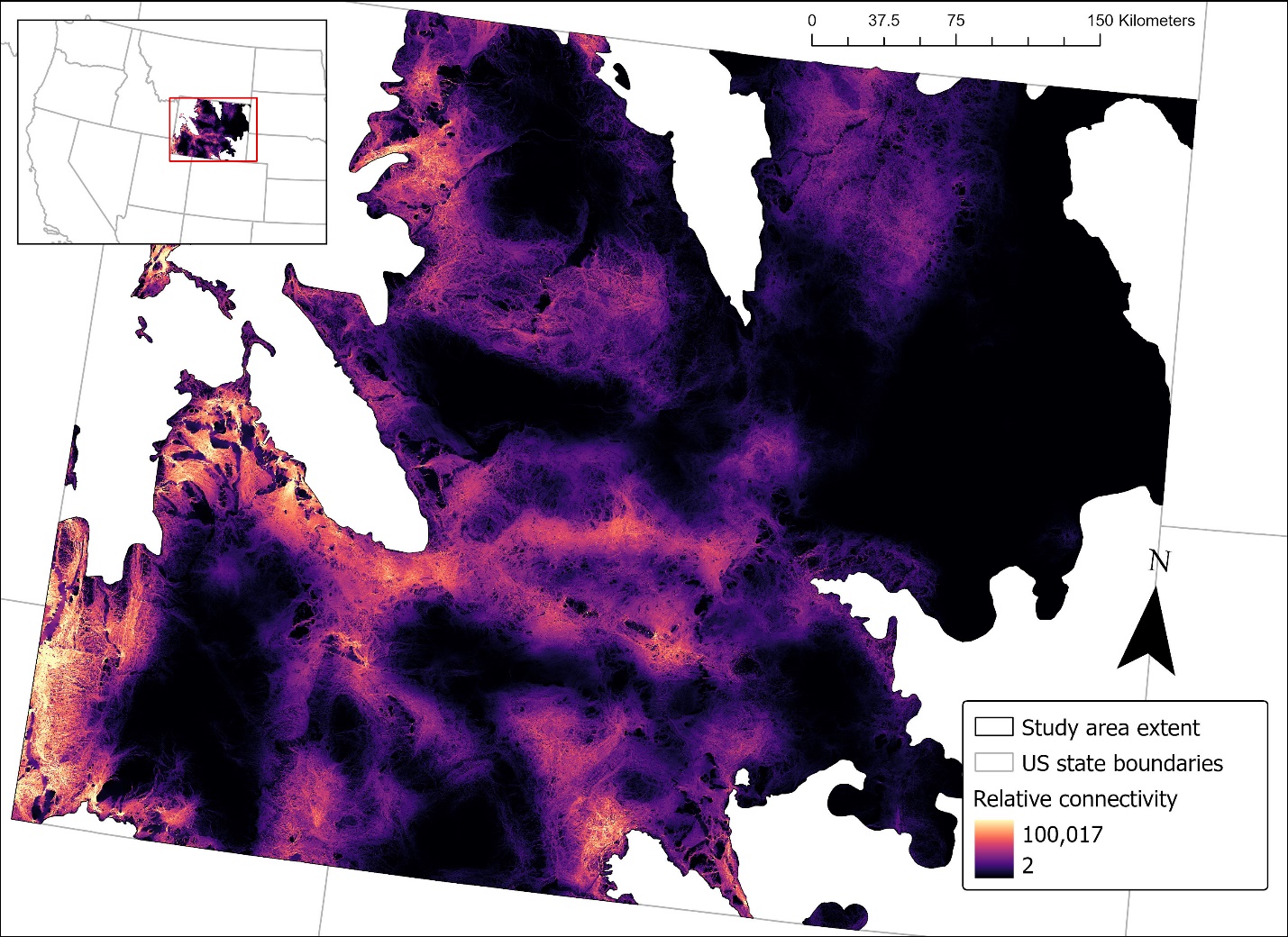


**Figure SA4:** 30-m resolution map of contemporary (2020) sagebrush connectivity across Wyoming, USA. Study area extent approximates the sagebrush biome within the state.

**
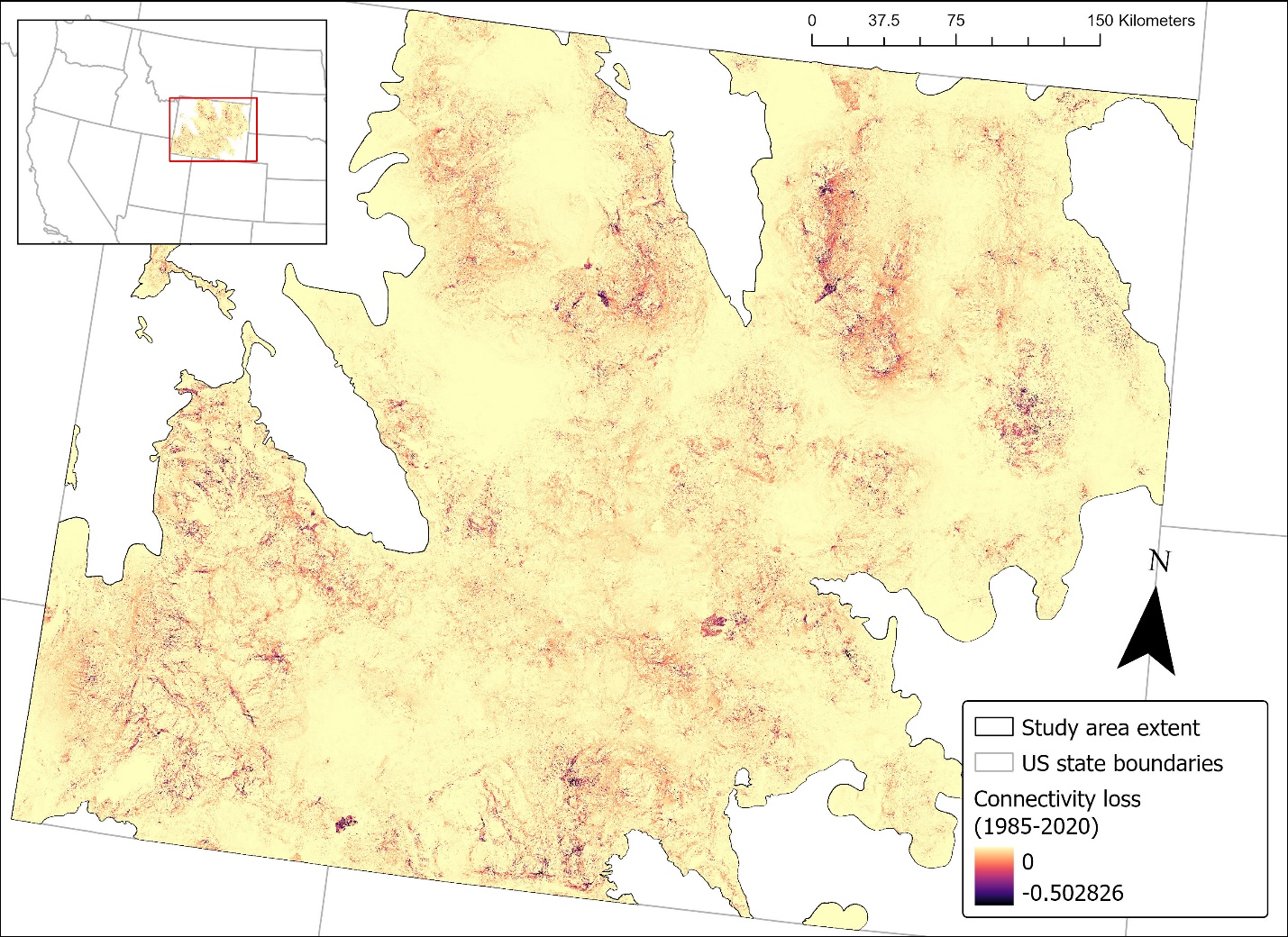
Figure SA5:** Map of greater sage-grouse lek connectivity loss (1985 to 2020) across Wyoming, USA. Study area extent approximates the sagebrush biome within the state.

**Sagebrush-obligate & sagebrush-associated songbird median densities**

Environmental variables incorporated in the density-habitat models included elevation, terrain ruggedness, maximum summertime Normalized Difference Vegetation Index (NDVI), Palmer Drought Severity Index, sagebrush cover, pinyon-juniper cover, cropland cover, annual herbaceous (i.e., annual exotic grass) cover, herbaceous cover, litter cover, linear disturbance (e.g., roadways and powerlines), and point disturbance (e.g., well pads; Van Lanen et al. 2023). Because some pixels within our study area had point disturbance values exceeding 130 standard deviations above mean values used to train the development models, we masked out pixels within the predicted density raster layers where point disturbance values exceeded the maximum point disturbance values in the training data. Additionally, we moderated remaining areas with high predicted densities by setting pixel values exceeding the 99% quantile of all pixel values to the 99% quantile of pixel values.

**Greater sage-grouse mean lek abundance**

Details on lek count protocol are summarized in Monroe et al. (2022), and we included both counts and surveys in our analyses. We modeled the observation process for lek count *y* at lek *i*, year *t*, and visit *j* with a binomial distribution, given latent population size *N_i,t_* and detection probability *p_i,t,j_*: *y_i,t,j_*|*N_i,t_* ~ Binomial(*N_i,t_*, *p_i,t,j_*). We fit covariates to the *p_i,t,j_* on the logit scale including linear and quadratic effects of minutes since sunrise, linear, quadratic, and cubic effects of ordinal date, linear effect of year, and an indicator for count type (count or survey), and a visit-specific random term (Monroe et al. 2019, 2022). Abundance in year *t* (*N_i,t_*) was a function of abundance and the annual finite rate of population change (λ) in year *t* – 1: *N_i,t_* ~ Poisson(*N_i,t−_*_1_ λ*_i,t_*_−1_). We allowed λ*_i,t_*_−1_ to vary with a random term for year (γ_t_), where γ_t_ ~ Normal(0, σ_year_), and annual terms to vary by population cluster *c*, with η*_c,t_* ~ Normal(γ_t_, σ_cluster_). We fit the model in a Bayesian framework using NIMBLE (v. 0.11.1, de Valpine et al. 2017) in R (R Core Team 2021). We sampled from three parallel chains for 30,000 iterations, thinning every 10^th^ iteration after discarding the first 7,500 iterations. We evaluated convergence by visually examining chains and calculating an $\hat{R}$ statistic (Gelman and Ruben 1992) where $\hat{R}$ <1.1 suggested apparent convergence. We used a sum-to-zero constraint for the annual random term to improve mixing of parallel chains (γ_t_; Ogle and Barber 2020). We also determined the model had adequate fit using a posterior predictive check (Bayesian p-value) based on chi-squared discrepancy between observed and predicted lek counts or surveys (Kéry and Schaub 2012). We used posterior samples from the state-space model to estimate the mean and CV across iterations for *N* for each lek in 2019. We then averaged means and CVs of *N* within each level 1 cluster.

**Methods for calibrating boundary penalties**

We used boundary penalties in problems 2a (high resilience), 3b (SCD resilience), and 3c (SCD connectivity) to promote clustering of selected restoration sites near intact sagebrush (Pyke 2012). Boundary penalties disincentivize the solver from selecting isolated pixels by increasing the cost of those pixels relative to the increase in total boundary length that selecting that pixel will cause (i.e., higher boundary penalty values will more severely penalize selection of isolated pixels). Because boundary penalties influence optimization relative to the values of the input layers used to inform the optimization problem, we calibrated boundary penalty selection by running multiple iterations of each of the above problems, iteratively increasing the boundary penalty by quarter orders of magnitude (i.e., 0.01, 0.05, 0.1, 0.5, 1.0, 1.5, 2.5, 5.0, 7.5, 10.0, 12.5, 50, 500). For each iteration, we plotted the total area of the solution, total cost (in terms of spring soil moisture availability or connectivity, respectively), and total feature layer values (for each focal species). We used these plots to identify where changes in boundary penalties had the most influence (i.e., near or below the asymptote where cost, feature, or area values plateaued) and then inspected the mapped solutions of those iterations for a desired degree of clustering near intact sagebrush sites (Ardron et al. 2010). For problem 2a (high resilience), we selected a boundary penalty of 1.0 for the all-species iteration, and then modified the boundary penalty for each single species iteration based on the proportional contribution of feature layer values from each species (i.e., 0.2 for pygmy rabbit, 0.06 for sagebrush sparrow, 0.14 for greater sage-grouse, 0.17 for sage thrasher, 0.19 for green-tailed towhee, and 0.24 for Brewer’s sparrow). For problem 3b (SCD resilience) we used a boundary penalty of 2.5 and for 3c (SCD connectivity) we used 1.5.

**REFERENCES**

Ardron, J. A., H. P. Possingham, and C. J. Klein. 2010. Marxan Good Practices Handbook, Version 2. Pacific Marine Analysis & Research Association, Victoria, BC, Canada.

Bezanson, J., A. Edelman, S. Karpinski, and B. Shah. 2017. Julia: A Fresh Approach to Numerical Computing. SIAM Rev **59**:65–98.

de Valpine, P., D. Turek, C. J. Paciorek, C. Anderson-Bergman, D. T. Lang, and R. Bodik. 2017. Programming With Models: Writing Statistical Algorithms for General Model Structures With NIMBLE. Journal of Computational and Graphical Statistics **26**:403-413.

Dickson, B. G., C. M. Albano, R. Anantharaman, P. Beier, J. Fargione, T. A. Graves, M. E. Gray, K. R. Hall, J. J. Lawler, P. B. Leonard, C. E. Littlefield, M. L. McClure, J. Novembre, C. A. Schloss, N. H. Schumaker, V. B. Shah, and D. M. Theobald. 2019. Circuit-theory applications to connectivity science and conservation. Conservation Biology **33**:239-249.

Falgout, J. T., J. Gordon, B. William, and M. J. Davis. 2022. USGS Advanced Research Computing, USGS Denali Supercomputer. U.S. Geological Survey.

Gelman, A., and D. B. Rubin. 1992. Inference from Iterative Simulation Using Multiple Sequences. Statistical Science **7**:457-472.

Hall, K. R., R. Anantharaman, V. A. Landau, M. Clark, B. G. Dickson, A. Jones, J. Platt, A. Edelman, and V. B. Shah. 2021. Circuitscape in Julia: Empowering Dynamic Approaches to Connectivity Assessment. Land **10**:301.

Kéry, M., and M. Schaub (Eds). 2012. Bayesian Population Analysis using WinBUGS: A hierarchical perspective. Academic Press, Waltham, MA, USA.

Landau, V., V. Shah, R. Anantharaman, and K. Hall. 2021. Omniscape.jl: Software to compute omnidirectional landscape connectivity. Journal of Open Source Software 6.

McRae, B. H., and P. Beier. 2007. Circuit theory predicts gene flow in plant and animal populations. Proc Natl Acad Sci **104**:19885-19890.

McRae, B. H., K. Popper, A. Jones, M. Schindel, S. Buttrick, K. R. Hall, R. S. Unnasch, and J. Platt. 2016. Conserving Nature’s Stage: Mapping Omnidirectional Connectivity for Resilient Terrestrial Landscapes in the Pacific Northwest. The Nature Conservancy, Portland, Oregon.

McRae, B. H., B. G. Dickson, T. H. Keitt, and V. B. Shah. 2008. Using circuit theory to model connectivity in ecology, evolution, and conservation. Ecology **89**:2712-2724.

Monroe, A. P., J. A. Heinrichs, A. L. Whipple, M. S. O’Donnell, D. R. Edmunds, and C. L. Aldridge. 2022. Spatial scale selection for informing species conservation in a changing landscape. Ecosphere **13**:e4320.

Monroe, A. P., G. T. Wann, C. L. Aldridge, and P.S. Coates. 2019. The importance of simulation assumptions when evaluating detectability in population models. Ecosphere **10**:e02791.

O’Donnell, M. S., D. R. Edmunds, C. L. Aldridge, J. A. Heinrichs, A. P. Monroe, P. S. Coates, B. G. Prochazka, S. E. Hanser, L. A. Weichman, T. J. Christiansen, A. A. Cook, S. P. Espinosa, L. J. Foster, K. A. Griffin, J. L. Kolar, K. S. Miller, A. M. Moser, T. E. Remington, T. J. Runia, L. A. Schreiber, M. A. Schroeder, S. J. Stiver, N. I. Whitford, and C. S. Wightman. 2021. Synthesizing and analyzing long-term monitoring data: A greater sage-grouse case study. Ecological Informatics **63**:101327.

Ogle, K., and J. J. Barber. 2020. Ensuring identifiability in hierarchical mixed effects Bayesian models. Ecological Applications **30**:e02159.

Pyke, D. A. 2012. Restoring and Rehabilitating Sagebrush Habitats. Pages 531-548 in S. T. Knick and J. W. Connelly, editors. Greater sage-grouse—Ecology and conservation of a landscape species and its habitats. University of California Press, Berkeley, CA, USA.

R Core Team. 2021. R: A language and environment for statistical computing. R Foundation for Statistical Computing, Vienna, Austria.

Rigge, M., D. Meyer, and B. Bunde. 2021. Ecological potential fractional component cover based on Long-Term satellite observations across the western United States. Ecological Indicators **133**.

Rigge, M., B. Bunde, K. Postma, and H. Shi. 2022. Rangeland Condition Monitoring Assessment and Projection (RCMAP) Fractional Component Time-Series Across the Western U.S. 1985-2021: U.S. Geological Survey data release, <https://doi.org/10.5066/P9ODAZHC>.

Van Lanen, N. J., A. P. Monroe, and C. L. Aldridge. 2023. Data and analytical code assessing eleven songbird species' responses to environmental change during summertime (2008 - 2020) in the InterMountain West, USA: U.S. Geological Survey data release.

***Disclaimer***

Any use of trade, firm, or product names is for descriptive purposes only and does not imply endorsement by the U.S. Government. This work was partly funded by the U.S. Department of Interior, Bureau of Land Management, in support of fuel break research.
