# Supplementary material for: Different Data for Different Goals: Exploring Trade‐Offs and Synergies in the Use of Spatial Data Inputs to Optimize Conservation Action in Sagebrush Ecosystems
Source: Ecol Evol. 2025 Sep 30;15(10):e72214. doi: 10.1002/ece3.72214 (PMC12483841; doi:10.1002/ece3.72214)
Supplement: Supplementary file 2 — Data S2: ece372214‐sup‐0002‐DataS2.docx. [file ECE3-15-e72214-s001.docx]

Supplemental B: Additional results from the manuscript ***“Different data for different goals: exploring trade-offs and synergies in the use of spatial data inputs to optimize conservation action in sagebrush ecosystems”***

Jessica E. Shyvers^1,2*^ (ORCID: 0000-0002-4307-0004), Bryan C. Tarbox^1^ (0000-0001-5040-3949), Adrian P. Monroe^1^ (0000-0003-0934-8225), Nicholas J. Van Lanen^1^ (0000-0003-0871-0261), Benjamin S. Robb^1,3^ (0000-0003-1419-3918), Erin K. Buchholtz^1,4^ (0000-0002-1985-9531), Courtney J. Duchardt^5^ (0000-0003-4563-0199), David R. Edmunds^1^ (0000-0002-5212-8271), Michael S. O’Donnell^1^ (0000-0002-3488-003X), Nathan D. Van Schmidt^1,6^ (0000-0002-5973-7934), Julie A. Heinrichs^7^ (0000-0001-7733-5034), Cameron L. Aldridge^1^ (0000-0003-3926-6941)

^1^U.S. Geological Survey, Fort Collins Science Center, 2150 Centre Ave, Bldg. C, Fort Collins, CO 80526

^2^The Nature Conservancy, Protect Oceans, Land and Water. Fort Collins, CO (current affiliation)

^3^Department of Zoology and Physiology, University of Wyoming, Laramie, WY (current affiliation)

^4^U.S. Geological Survey, South Carolina Cooperative Fish and Wildlife Research Unit, Clemson University, Clemson, SC (current affiliation)

^5^School of Natural Resources and the Environment, University of Arizona, Tucson, AZ 85721 (current affiliation)

^6^San Francisco Bay Bird Observatory, 524 Valley Way, Milpitas, CA 95035 (current affiliation)

^7^Natural Resource Ecology Laboratory, Colorado State University, Fort Collins, CO 80523 in cooperation with the U.S. Geological Survey, Fort Collins Science Center, 2150 Centre Ave, Bldg. C, Fort Collins, CO 80526

*Corresponding Author: jess.shyvers@tnc.org, 970-556-8119

**Summary statistics for comparing sites selected under different prioritization problems**

**Table SB1:** Total area (ha) of all sites selected by each prioritization problem, degraded and intact sagebrush sites, Sagebrush Conservation Design (SCD) core sagebrush (CSA) and growth opportunity areas (GOA), and Wyoming’s greater sage-grouse core areas.

| Problem/conservation area | # of planning units | Hectares |
| --- | --- | --- |
| 1a (no cost; greater sage-grouse) | 6,092,882 | 548,359.4 |
| 1a (no cost; pygmy rabbit) | 3,019,782 | 271,780.4 |
| 1a (no cost; Brewer’s sparrow) | 3,273,838 | 294,645.4 |
| 1a (no cost; green-tailed towhee) | 2,287,887 | 205,909.8 |
| 1a (no cost; sagebrush sparrow) | 908,599 | 81,773.9 |
| 1a (no cost; sage thrasher) | 2,385,746 | 214,717.1 |
| 1a (no cost; all species) | 6,104,835 | 549,435.2 |
| 1b (connectivity) | 6,192,189 | 557,297 |
| 1c (future sagebrush) | 6,255,069 | 562,956.2 |
| 1d (low resiliency; no mask) | 6,682,714 | 601,444.3 |
| 1d (low resiliency; >5% future sagebrush) | 6,513,116 | 586,180.4 |
| 1d (low resiliency; >10% future sagebrush) | 6,364,758 | 572,828.2 |
| 2a (high resiliency) | 2,157,189 | 194,147 |
| 2b (uncertainty; low) | 321,200 | 28,908 |
| 2b (uncertainty; medium) | 289,834 | 26,085.06 |
| 2b (uncertainty; high) | 1,024,394 | 92,195.46 |
| 3a (SCD protection) | 5,154,844 | 463,936 |
| 3b (SCD resiliency) | 820,450.6 | 73,840.55 |
| 3c (SCD connectivity) | 890,150.9 | 80,113.58 |
| Intact sagebrush | 33,640,752 | 3,027,668 |
| Degraded sagebrush | 9,242,648 | 831,838.3 |
| SCD CSAs | --- | 6,620,684 |
| SCD GOAs | --- | 5,839,811 |
| Wyoming’s greater sage-grouse core areas | --- | 6,260,784 |

*Problem set #1: Identifying priority protection sites*


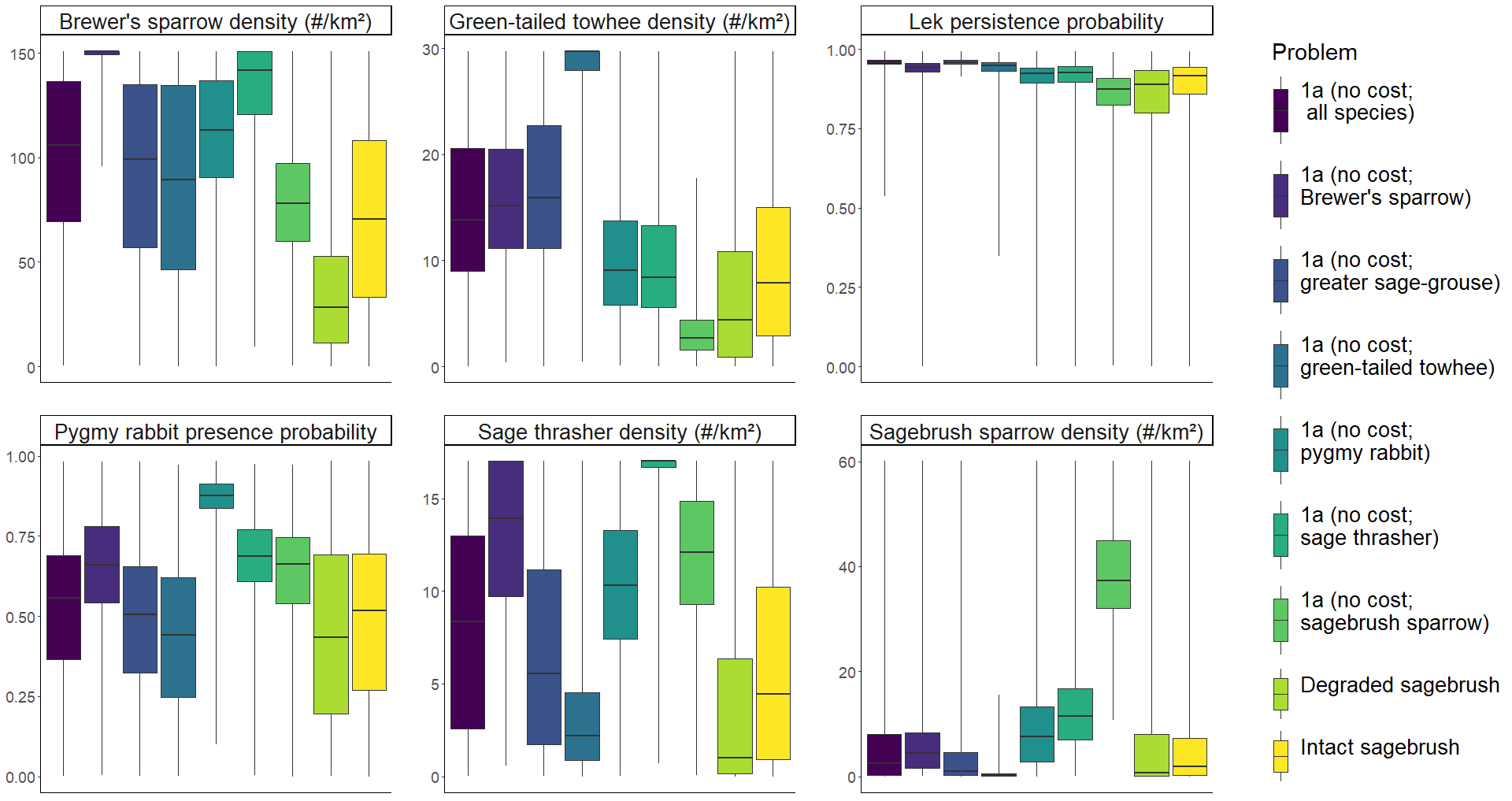


**Figure SB1:** Boxplots representing medians (black line), 25%-75% quartiles (box), and minimums and maximums (whiskers) of focal species’ feature layer values (e.g., densities) within sites selected by optimization problem 1a (no cost) to prioritize protection of Brewer’s sparrow, greater sage-grouse, green-tailed towhee, pygmy rabbit, sage thrasher, sagebrush sparrow, or all six species in sagebrush sites in Wyoming, USA. Each panel depicts values of a single feature layer (e.g., lek persistence probability) across multiple optimization problems (e.g., 1a (no cost; all species)). Boxplots of each feature layer value within all degraded and intact sagebrush sites are also illustrated for reference.

**
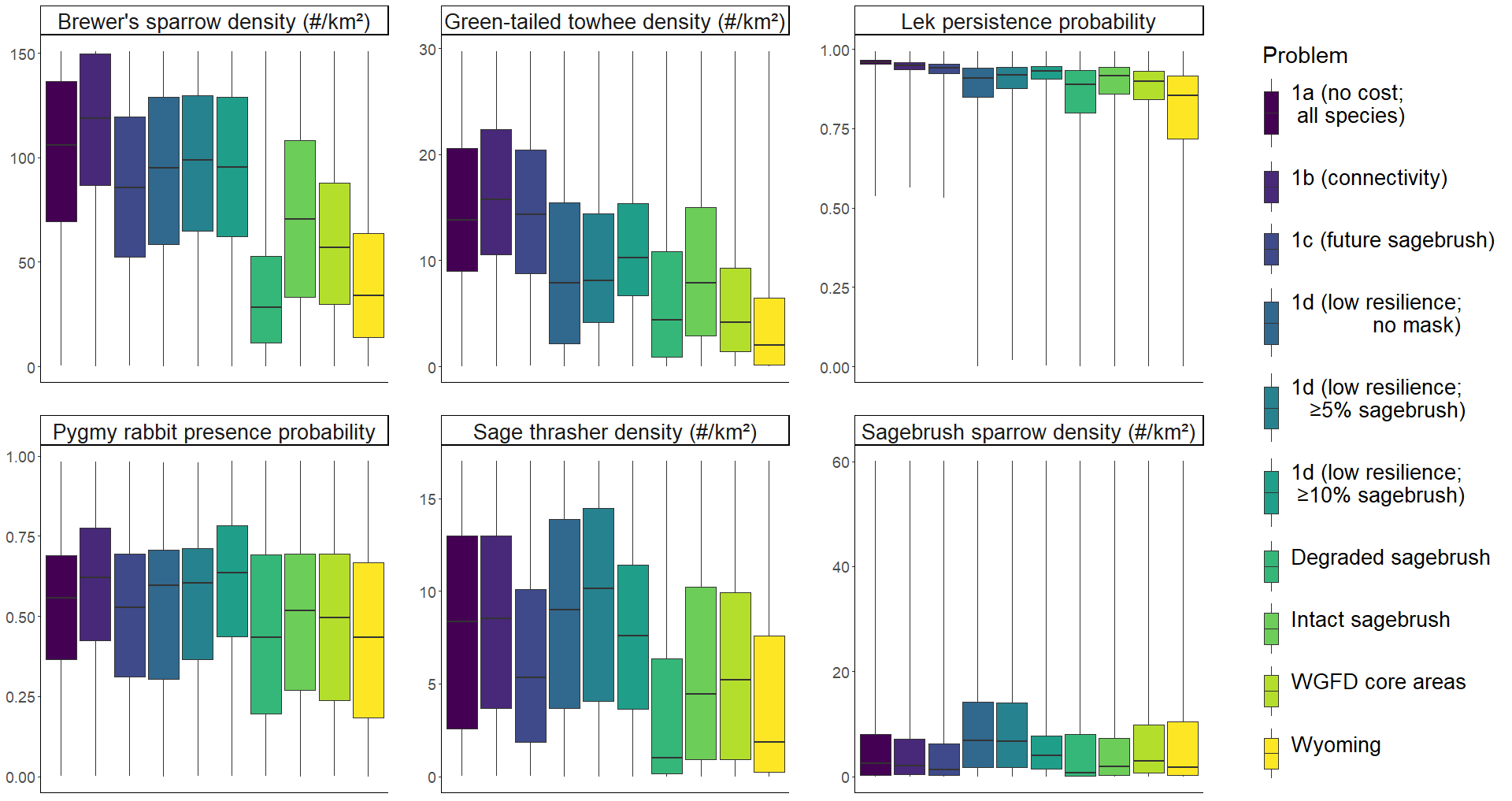
**

**Figure SB2:** Boxplots representing medians (black line), 25%-75% quartiles (box), and minimums and maximums (whiskers) of focal species’ feature layer values (e.g., densities) within sites selected by optimization problem set 1 to identify priority protection sites for all focal species while a) not considering any cost, b) maximizing sagebrush connectivity, c) maximizing future sagebrush cover, or d) minimizing spring soil moisture availability. Each panel depicts values of a single feature layer (e.g., lek persistence probability) across multiple optimization problems (e.g., 1a (no cost; all species)). Boxplots of each feature layer value within degraded sagebrush, intact sagebrush, Wyoming’s greater sage-grouse core areas, and Wyoming’s sagebrush biome (Wyoming) are also illustrated for reference.


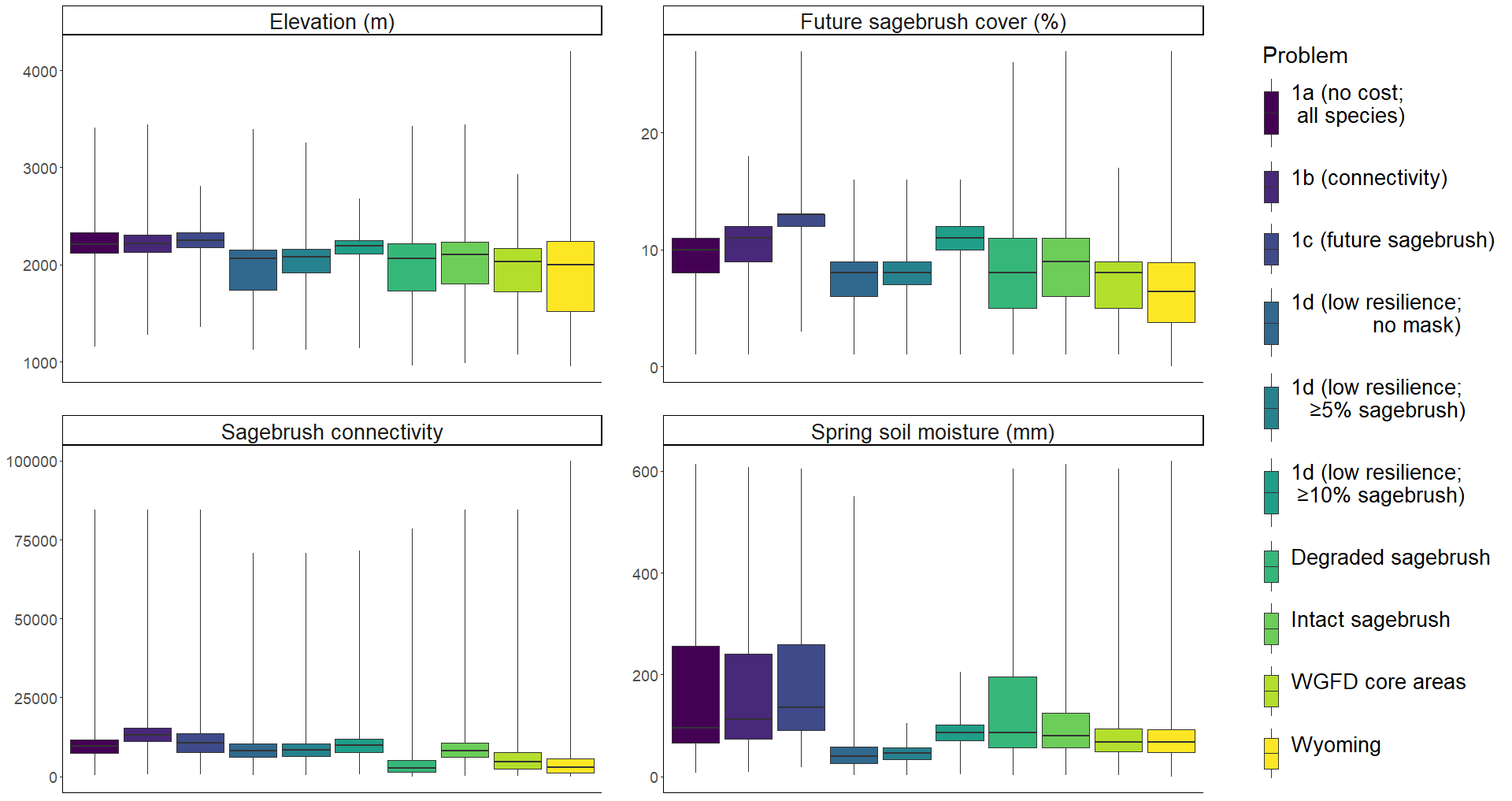


**Figure SB3:** Boxplots representing medians (black line), 25%-75% quartiles (box), and minimums and maximums (whiskers) of cost layer values (e.g., future sagebrush cover) within sites selected by optimization problem set 1 to identify priority protection sites for all focal species while a) not considering any cost, b) maximizing sagebrush connectivity, c) maximizing future sagebrush cover, or d) minimizing spring soil moisture availability. Each panel depicts values of a single cost layer (e.g., sagebrush connectivity) across multiple optimization problems (e.g., 1a (no cost; all species)). Elevation is also represented in one panel for reference, as it is correlated with some cost layers such as future sagebrush cover and spring soil moisture availability. Boxplots of each cost layer value within degraded sagebrush, intact sagebrush, Wyoming’s greater sage-grouse core areas, and Wyoming’s sagebrush biome (Wyoming) are also illustrated for reference.

*Problem set #2: Identifying priority restoration sites*


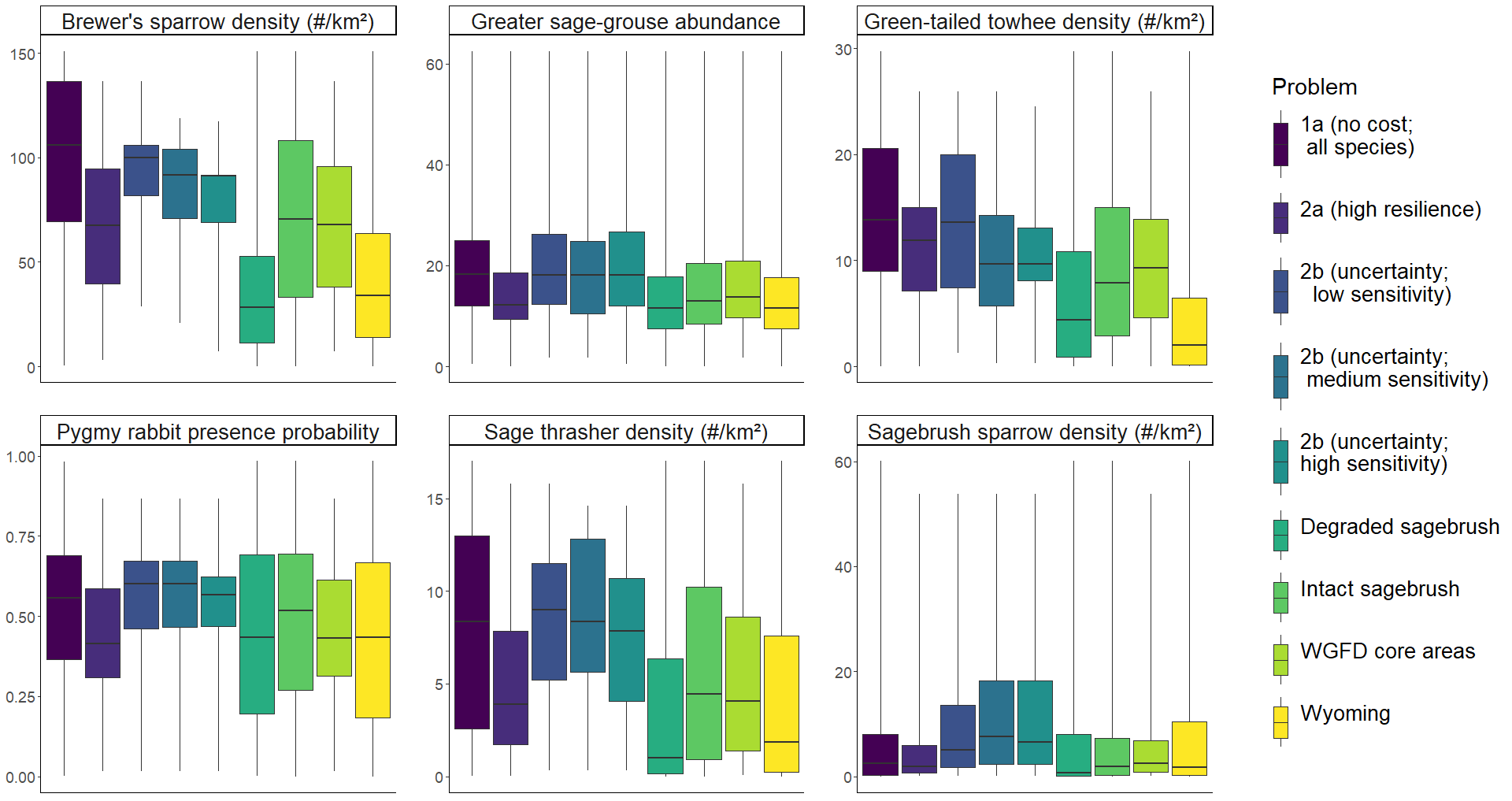


**Figure SB4:** Boxplots representing medians (black line), 25%-75% quartiles (box), and minimums and maximums (whiskers) of focal species’ feature layer values (e.g., densities) within sites selected by optimization problem set 2 to identify priority restoration sites for all focal species while a) maximizing spring soil moisture availability or b) assessing data uncertainty. Each panel depicts values of a single feature layer (e.g., greater sage-grouse abundance) across multiple optimization problems (e.g., 2a (high resilience)). For all species besides greater sage-grouse, feature layers used in problem set 2 represent potential habitat values based on average values across intact sagebrush within level 1 management cluster polygons (refer to Methods for details). Boxplots of each feature layer value selected by problem 1a (no cost), and within degraded sagebrush, intact sagebrush, Wyoming’s greater sage-grouse core areas, and Wyoming’s sagebrush biome (Wyoming), are also illustrated for reference.


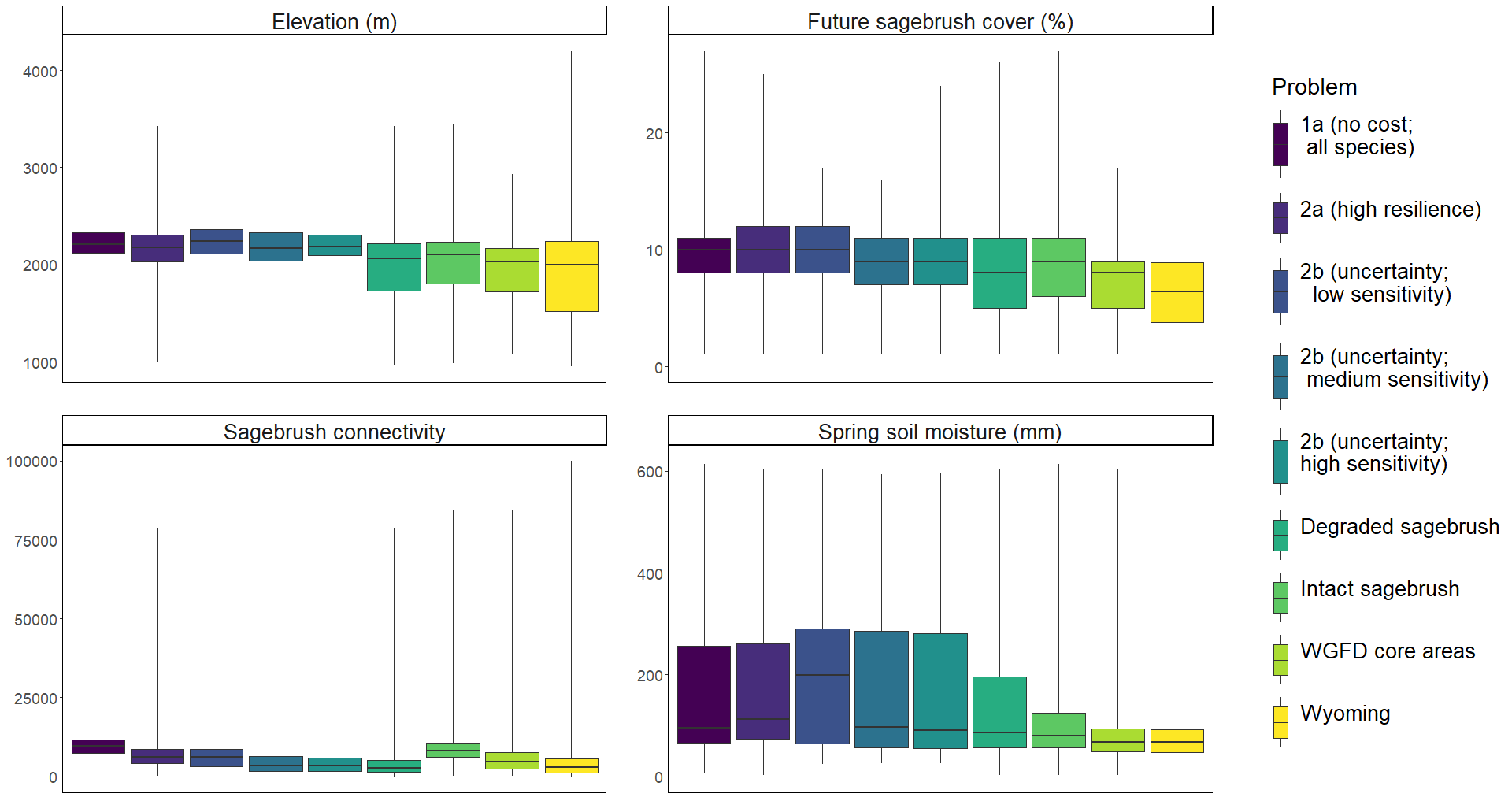


**Figure SB5:** Boxplots representing medians (black line), 25%-75% quartiles (box), and minimums and maximums (whiskers) of cost layer values (e.g., future sagebrush cover) within sites selected by optimization problem set 2 to identify priority restoration sites for all focal species while a) maximizing spring soil moisture availability or b) assessing data uncertainty. Each panel depicts values of a single cost layer (e.g., sagebrush connectivity) across multiple optimization problems (e.g., 2a (high resilience)). Elevation is also represented in one panel for reference, as it is correlated with some cost layers such as future sagebrush cover and spring soil moisture availability. Boxplots of each cost layer value selected by problem 1a (no cost), and within degraded sagebrush, intact sagebrush, Wyoming’s greater sage-grouse core areas, and Wyoming’s sagebrush biome (Wyoming), are also illustrated for reference.

*Problem set #3*: *Enhancing existing conservation strategies*

**
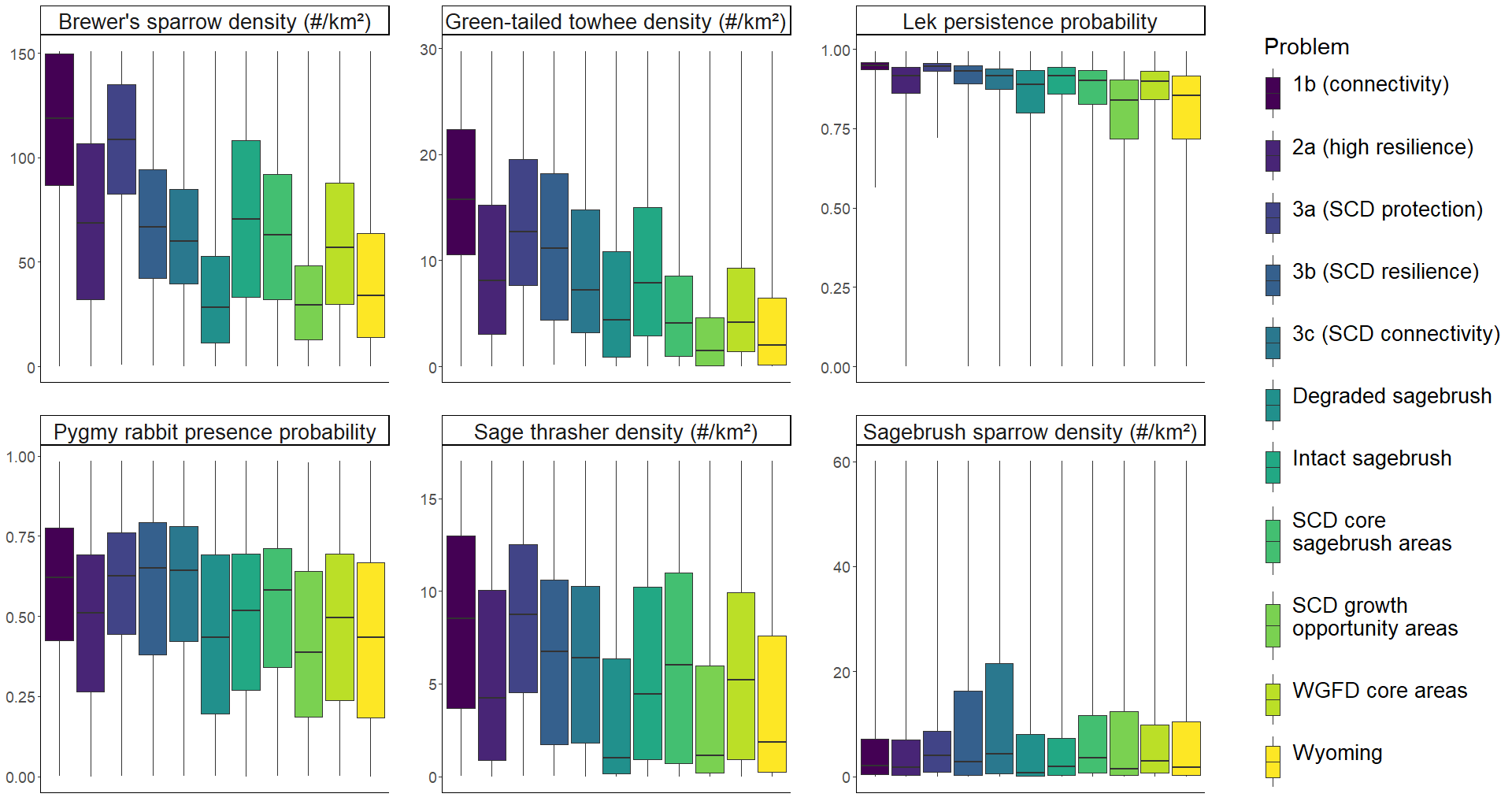
**

**Figure SB6:** Boxplots representing medians (black line), 25%-75% quartiles (box), and minimums and maximums (whiskers) of focal species’ feature layer values (e.g., densities) within sites selected by optimization problem set 3 to enhance existing conservation strategies by a) protecting focal species’ habitat and lek connectivity within core sagebrush and growth opportunity areas, b) restoring focal species’ habitat where success is most likely within core sagebrush and growth opportunity areas, or c) restoring focal species’ habitat and lost lek connectivity within core sagebrush and growth opportunity areas. Each panel depicts values of a single feature layer (e.g., lek persistence probability) across multiple optimization problems (e.g., 3a (SCD protection)). Boxplots of each feature layer value selected by problems 1a (no cost) and 2a (high resilience), as well as within degraded sagebrush, intact sagebrush, Sagebrush Conservation Design (SCD) core sagebrush and growth opportunity areas, Wyoming’s greater sage-grouse core areas, and Wyoming’s sagebrush biome (Wyoming), are also illustrated for reference.


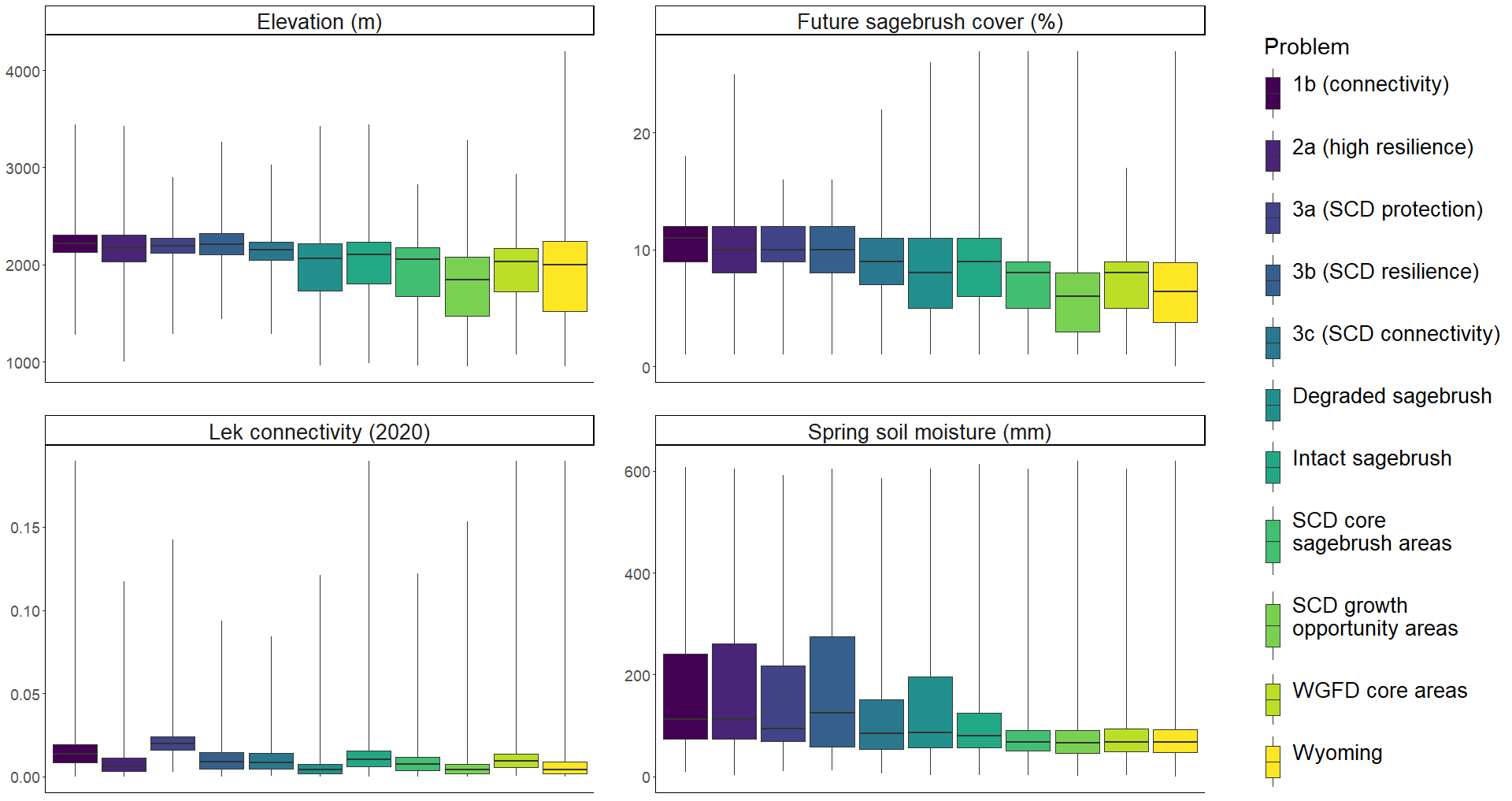


**Figure SB7:** Boxplots representing medians (black line), 25%-75% quartiles (box), and minimums and maximums (whiskers) of cost layer values (e.g., future sagebrush cover) within sites selected by optimization problem set 3 to enhance existing conservation strategies by a) protecting focal species’ habitat and lek connectivity within core sagebrush and growth opportunity areas, b) restoring focal species’ habitat where success is most likely within core sagebrush and growth opportunity areas, or c) restoring focal species’ habitat and lost lek connectivity within core sagebrush and growth opportunity areas. Each panel depicts values of a single cost layer (e.g., lek connectivity) across multiple optimization problems (e.g., 3a (SCD protection)). Elevation is also represented in one panel for reference, as it is correlated with some cost layers such as future sagebrush cover and spring soil moisture availability. Boxplots of cost layer values within selected pixels for problems 1a (no cost) and 2a (high resilience), as well as within degraded sagebrush, intact sagebrush, Sagebrush Conservation Design (SCD) core sagebrush and growth opportunity areas, Wyoming’s greater sage-grouse core areas, and Wyoming’s sagebrush biome (Wyoming), are also illustrated for reference.

***Disclaimer***

Any use of trade, firm, or product names is for descriptive purposes only and does not imply endorsement by the U.S. Government. This work was partly funded by the U.S. Department of Interior, Bureau of Land Management, in support of fuel break research.
